# Biomimetic UV photo-protection of skin surface by structured epicuticular wax films

**DOI:** 10.1016/j.mtbio.2025.101991

**Published:** 2025-06-17

**Authors:** Anuja Das, Luca Polacchi, Jean-Yves Fouron, Antoine Montaux-Lambert, Laurent Billon, Gustavo S. Luengo

**Affiliations:** a Bio-Inspired Materials Group: Functionalities & Self-Assembly, Université de Pau & Pays Adour, CNRS, IPREM UMR 5254, Technopole Hélioparc, 2 avenue Angot, PAU cedex 09, 64053, PAU, France; b L’Oréal Research & Innovation, 1 Av. Eugène Schueller, Aulnay-sous-Bois, 93600, Paris, France; c L’Oréal Research & Innovation, 100 Av. de Stalingrad, Chevilly-Larue, 94550, France

**Keywords:** Biomimicry, Epicuticular wax, Surface structuration, Skin surfaces, UV-Reflectance

## Abstract

The realms of biomimicry encourage us to explore and replicate the remarkable functionalities found in the big variety of living organisms such as plants, birds, animals etc. Inspired by the ultraviolet (UV) reflective characteristics of self-assembled epicuticular wax of plant leaves, in this article, we present a biomimetic plant-inspired approach to pattern the surface of skin with wax coatings and enhance its UV resistance. Through a physico-chemical approach, we coat chemically homogenous (as well as heterogenous) chemical composition of waxes from its solution on quartz substrate. By controlling the self-assembly conditions, diverse surface morphologies are obtained with *Euphorbia cerifera* (commonly known as *Candelilla*, chemically heterogenous wax) and *Myristyl Palmitate* (present in *Phytolacca Acinosa*, chemically homogenous alkyl esters wax). Optical measurements show increased reflectance in visible spectra for *Candelilla* wax coatings exhibiting globules, plate-like crystalline structures at the surface which contributes to higher roughness parameters. With homogenous wax, maximum reflectance is obtained for dual scale morphology which includes self-assembled 3D plate-like structures at an optimum length-scale. Our experiments reveal that the combined effect of vertically and horizontally placed stacks of crystal plates at micron and sub-micron scales induces maximum scattering effect. This geometrical organisation effectively decreases transmission of incident radiations to the underlying surface leading to enhanced photo-protection. Further, to showcase the feasibility of such approach for potential cosmetic applications, we replicate best performing structures on a commonly used model skin surface for UV absorption evaluation (polymethyl methacrylate plates) and on real *ex-vivo Stratum Corneum,* the outermost layer of skin. For realistic substrates, scattering effect is additionally dependent on nature of intrinsic substrate patterns and roughness in which case the feature height (**h_F_**) of 3D structures should be greater than substrate patterns to achieve maximum reflectance. This study highlights the formation of physical structuration with biomaterials and presents insights on scattering induced by such plant-based structures with potential for dermatological or cosmetic applications.

## Graphical abstract

**Figure:**
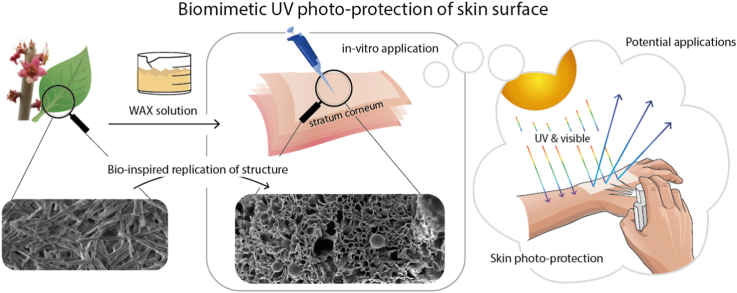

## 1 Introduction

Most living organisms in Nature develop innate abilities to adapt to their habitat and sustain adverse conditions. Exposure to UV radiation is one such condition that has detrimental consequences on all type of living organisms. For example, in plants, UV radiation causes severe damage to its organs, most notably destruction of enzymes and deactivation of DNA, *i.e.* biomacromolecules [1]. Plants possess natural strategies to absorb useful part of sunlight for photosynthesis and restrict penetration of harmful UV radiation. One such ability is formation of waxy layer, commonly known as *Epicuticular Wax*, on the outermost surface of external organs. Epicuticular wax is produced *in-situ* by plants through complex biochemical pathways which forms a thin crystallized layer on the surface by self-assembly [2], leading to myriads of surface patterns [3]. Chemical composition of such wax depends on the plant species, type of habitat and organ it protects [4]. While most of the functionalities of the wax layer are attributed to chemical composition, physical structuration is also known to influence properties like UV-reflectivity and wettability [5]. Structure induced optical effects[[6], [7], [8]] and Superhydrophobicity [9] are well known phenomenon, most commonly observed as the iridescent colour displayed by bird's feather [7,8], butterfly wings [6] and superhydrophobic nature of lotus leaf [9]. These interfacial properties are primarily governed by the length scale of the physical structuration [7,10]. Similarly, in plants, physical structuration due to self-assembled epicuticular wax film provides functionalities like Superhydrophobicity and UV-reflectivity [[11], [12], [13], [14], [15]]. The harmful consequences to UV exposure are not restricted to plants and animals. Human skin, and more specifically the *Stratum Corneum* as the most superficial tissue of the epiderma, is also prone to UV radiations leading to compromised skin properties and diseases [16,17]. Therefore, techniques for UV photo-protectivity have continuously evolved over the years. Currently, most effective and widely used photo-protection mechanism is based on chemical defence, where several organic and/or inorganic UV-filtering compounds such as avobenzone, titanium dioxide (TiO_2_), zinc oxide (ZnO) are used as active ingredients for nullifying UV radiation reaching the epidermal layer of human skin [18,19]. However, active research is ongoing to shift to more bio-inspired solution by mimicking natural models which can either replace or work in conjunction with existing mechanism for enhancement of UV photo-protection [20,21]. Recently, Nature models have inspired development of several functional coatings by adopting approaches like direct replication on relevant materials, template assisted assembly or coating on patterned substrates [[22], [23], [24], [25], [26], [27]], direct-writing for patterned structural arrays [[28], [29], [30], [31], [32]], and several other methods [[33], [34], [35], [36], [37]] focussing on improving and developing sensory devices, anti-counterfeiting technique [30], smart coatings [38] and other optical applications [[35], [36], [37],^39^].

Conversely, replication of natural patterns on substrates of cosmetic interest faces a different challenge due to intrinsic roughness of these surfaces. While most of the above-mentioned approaches are straightword and reproducible, such techniques have severe limitations as patterned molds or direct writing techniques are not relevant for cosmetic surfaces like hair or skin. In the case of hair, the scales are sequentially superposed and tilted at a specific angle influencing both optical and tribological effects. This particular “*sawtooth*” like pattern has its advantages that may disappear when a new pattern or structure emerges [40]. For skin, the *microrelief* is the natural arrangement of tension lines on the surface which are associated to mechanoreceptors of the underneath dermal layers enabling tactile perception [41]. Besides, topography due to microrelief is crucial for controlling its tribological behaviour. Consequently, patterning of skin surface becomes challenging as it requires feasible approach for superposition of new patterns on existing microreliefs in order to retain skin's properties at micro and nano scale. In this context, previously, Jin et al. have shown patterning using mechanical imprinting [42], polymer coatings [43] and subsequently established variation in tribological properties of skin's outermost surface, *i.e. Stratum Corneum.* [42,43].

In this article, we present a facile, versatile and biomimetic plant-based approach to structure skin surfaces following evaporative induced self-assembly of waxes, inspired by epicuticular waxes on plant leaves. This physico-chemical approach closely resembles topical application of formulation from cosmetic perspective that is based on evaporation of a wax solution in ambient situations. In previous article reported by our group, the fundamentals of the approach was thoroughly investigated and reported [44]. Here, we explore in detail the UV scattering ability due to physical structuration by coating both absorbing and non-absorbing wax films from its solution in different solvents (solvent mixtures) on quartz plates. Thereafter, optical functionality of these biomimetic films is established by correlating surface topography with their UV-reflectance. Further, to determine the feasibility of this approach for skin-care application, we replicate high performing structures on model skin substrate like PMMA plates (smooth and textured), and subsequently, show application of this approach for *in* *vitro* patterning of real skin surface, the *Stratum Corneum*. For the first time, our results highlight the contribution of epicuticular waxes based surface structuration on UV reflectance of substrates and especially *in vitro* surface with *Stratum Corneum*. This provides insights on mimicking natural patterns on surfaces of cosmetic interest which can enhance UV photo-protectivity.

## 2 Materials and methods

### 2.1 Materials

*Candelilla* (CD), a natural plant wax was purchased from Multiceras, Mexico. Synthetic alkyl ester wax comprises myristyl stearate: myristyl palmitate at 60:40 ratio, which for the sake of simplicity will be denoted as *Myristyl Palmitate* (MP) wax. This was acquired from L'Oreal (Paris) delivered by different certified suppliers. Common solvents such as IsoPropyl Alcohol (*IPA*), n-hexanol (*hex*), tert-pentanol (*tPA*), cyclohexane (*CH*) were purchased from *Sigma Aldrich* and *TCI Chemicals*. Cosmetic grade solvents such as Isododecane (2,2,4,6,6-pentamethylheptane, *isoDD*), Isohexadecane (2,2,4,4,6,8,8 heptamethylnonane, *isoHD*) were provided by L’Oréal.

Here, we would like to highlight that the choice of wax was mainly driven by cosmetic applicative aspects. Similar work can be replicated with other waxes. *Candelilla* and *Myristyl Palmitate* are widely utilized in cosmetic formulations albeit for different properties [45].

### 2.2 Substrate specifications

- ***Quartz plates:*** Fused quartz microscopic slides of dimension 25 x 25 × 1.0 mm were acquired from *ThermoFisher Scientific.*
- **PMMA Plates:** Sandblasted PMMA plates of dimensions 50 mm × 50 mm x 6 μm were acquired from *Helioscreen.*
- ***Stratum Corneum* (SC):** 15–25 μm thick SCs were isolated *ex vivo* from human skin. These were obtained from human abdominal skin residues following plastic surgery, in accordance with ethical principles stated in the Helsinki declaration [42,46].

Surface characterization of the substrates and the roughness parameters are mentioned in section S2 of ESI.

### 2.3 Properties of wax

***The****r****mal analysis using Dynamic Scanning Calorimetry (DSC):*** Thermal analysis of the waxes was carried out using a DSC 3 STARe System Mettler Toledo. 2–6 mg of wax samples were added and sealed in aluminium pan of 40 μL in presence of air flow (50 ml/min), which were then allowed for two heating and cooling cycles. The samples were heated to 150 °C at 20 °C/min and cooled at 5 °C/min. Thermal parameters corresponding to start and end of melting, melting enthalpy, were calculated by analysing the curves using the equipment software, STARe Software (V16.40).

### 2.4 Optical characterization of raw wax materials by UV–vis Spectroscopy

Absorption bands of the raw wax materials were confirmed by measuring absorbance for UV and visible spectra at room temperature using *Shimadzu UV 2450* spectrometer. Candelilla wax was solubilized in a solvent mixture of isododecane: isopropanol (*isoDD:IPA*; 90:10 v/v) whereas *Myristyl Palmitate* was solubilized in IPA, both solubilized at ***C*_*n*_** ∼ 1.0 % w/v. Corresponding solvents (solvent mixtures) were used as reference for measurements.

### 2.5 Preparation and casting of wax solutions

Solutions of CD and MP were prepared by adding wax beads in different solvents/solvent mixtures (such as *isoDD: IPA*, *IPA:CH*) and heating the solution to a temperature above 65 °C (melting point of both waxes obtained from DSC measurements in Fig. S1 of ESI). At this temperature, the wax melts, and dissolves completely in the solvent. After complete dissolution, the solubilized wax solution was cooled down to room temperature. Homogenous wax solution of different concentration ***C*_*n*_** (w/v) were prepared. Prior to film casting, the solutions were thoroughly shaken for few minutes to ensure homogeneity. Before casting of wax solutions, surfaces of dry quartz plates were cleaned and modified by UV-Ozone cleaner (*UVO CLEANER, Model No. 42–220, Jelight Company Inc., USA*) to ensure complete spreading of solutions covering the entire substrate. PMMA plates were used as acquired and thin SC was placed on quartz plates which acted as base substrate during coating. Wax solution was casted by spin-coating (*Polos 200, SPS-International*) at a spin speed of 500 r.p.m for a duration of 30 s. Samples after spin coating were allowed to dry at ambient conditions and temperature of 25 °C.

### 2.6 Surface characterization

The surface of the bare substrates, wax coated substrates was imaged and characterized by the following microscopic techniques which revealed structuration at length scale from millimetres to nanoscale. A minimum of three areas were imaged for each substrate at the centre to eliminate variation due to edge-effect.

- **Optical Microscopy (OM):** All wax coated and uncoated substrates were first observed under optical microscope with Leica (DMLM) in reflective mode to determine large area surface coverage and uniformity of the coated wax films. With optical microscope, 2D visualization at length scale from few millimetres to hundreds of microns is obtained.
- **Scanning Electron Microscopy (SEM):** To obtain surface morphology, we imaged completely dried films at length scale ranging from few hundreds to few microns with APERO-2 Scanning Electron Microscope (SEM, *ThermoFisher Scientific*). Prior to mounting in SEM, samples were coated with platinum films of thickness ∼4 nm and were charged at a voltage of 2 kV. All samples were imaged at ambient conditions.
- **Optical Profilometry:** Surface roughness and its parameters were determined by scanning the surfaces by Optical Profilometry in non-contact mode composed of controller CCS prima (*STIL SA, France*), a CHR80 sensor (with maximum vertical displacement of 80 μm) and displacement table (*Micromesure, STIL SA, France*) controlled with SurfaceMap data acquisition software (*DigitalSurf, France*). Raw data were rendered using MountainMap 9.0 (*DigitalSurf, France*).

Surface roughness parameters were obtained from analysis of raw data from optical profilometer scans of the samples. Roughness was defined by the following parameters [44]:

**H**: thickness of the continuous layer of wax film.

**h_F-avg_**: vertical distance of 3D structures on surface.

**SDQ** (root-mean-square-gradient): sharpness of the structures.

**SAL** (auto-correlation length): density of the 3D structures.

Details of surface roughness parameters and the physical implications are mentioned in section S2 and S3 of ESI.

The film thickness of structured wax films (**H**) was determined by measurements with optical profilometer across a scratch made carefully with a sharp needle (essentially that removes wax till the substrate). Details in this regard in provided in section S4 of ESI.

- **Atomic Force Microscopy (AFM):** Films were imaged using an atomic force microscope (Veeco diInnova, Bruker) in tapping mode with Si doped AFM probes from Bruker (Model: RTESP – 150). With AFM imaging, we identify surface morphology and the roughness of the films at sub-micron length scale. Raw AFM data were processed using open licensed software, *Gwyddion.*

### 2.7 Properties of wax films

- ***UV–Visible*** ***Spectroscopy***

The total transmission and reflectance of the samples were measured with a *Perkin-Elmer 860 Spectrophotometer* equipped with a 150 mm Labsphere diffuse integrating sphere with two ports (reflectance with the holder in the bottom horizontal position, and transmission) and a PMT/Pbs detector. A top access port allowed to view the beam position during alignment. The spectra were recorded at room temperature in steps of 1 nm at 266 nm. s^−1^ in the range 250–700 nm with a bandwidth of 4 nm. The instrument was calibrated with a certified Spectralon white standard *(Labsphere, North Sutton, USA*). A schematic of the instrument setup in total transmission and reflectance mode is shown in Scheme S2 of ESI.

- ***Surface******wettability:***

Wettability of wax coated quartz plates, PMMA plates and SC were acquired by Static Contact Angle Method using custom made optical tensiometer. 7 μL of water was dispensed for each measurement on horizontally placed samples. The images were analysed using software, developed at IPREM, integrated with the instrument. The analysis is performed by software-aided fitting procedure that finds the edges of drop and air-liquid interface (region of interest is defined by user) to determine the left and right contact angle. The contact angle reported in the article is an average of the contact angles generated for three areas on a specific sample. All measurements were performed at ambient conditions and temperature.

## 3 Results and discussion

### 3.1 Structuration on quartz plate

We follow one-step biomimetic approach to obtain structured wax films with Candelilla (chemically heterogenous natural wax) extracted from *Euphorbia cerifera* and Myristyle Palmitate (alkyl esters wax) present in *Phytolacca Acinosa* on quartz plate, first [47]. The waxes were solubilized in relevant solvent (solvent mixtures) and deposited on substrates at ambient conditions as reported in our previous study [44].

**Fig. 1:**
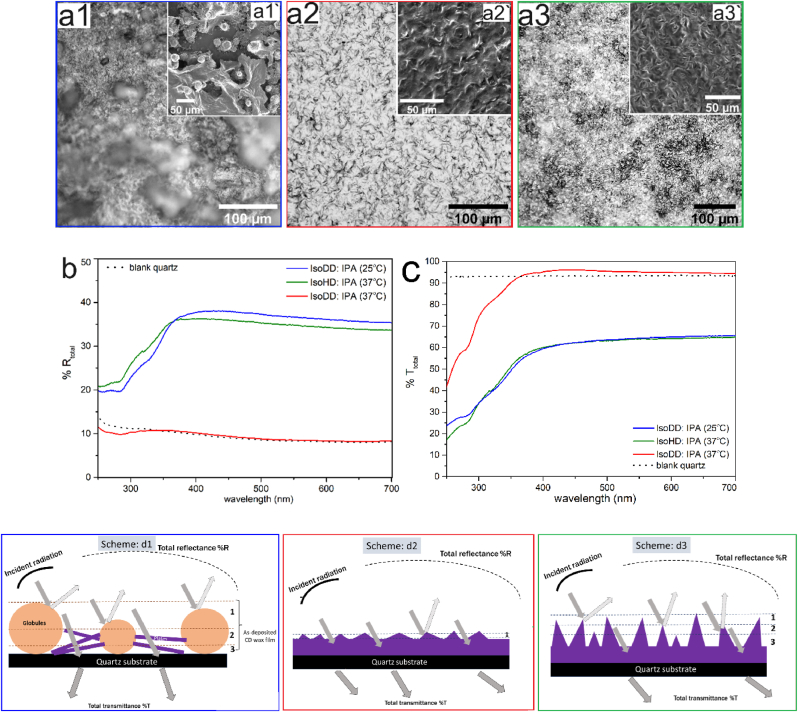
Surface morphologies obtained from deposition of CD wax from its solution in (**a1 – a3**) *isoDD:IPA* (90:10 v/v) at 25 °C; *isoDD:IPA* (90:10 v/v) at 37 °C and *isoHD:IPA* (90:10 v/v) at 37 °C respectively. Inset images show corresponding surface morphologies of the as-deposited films, imaged by Scanning Electron Microscope (SEM). Graphical representation showing total optical (**b**) reflectance (**%R_total_**) and (**c**) transmittance (**%T_total_**) for structured CD wax films. *Solid blue, green and red lines represent****%T_total_****and****%R_total_****for films obtained from isoDD:IPA (25°C); isoHD:IPA (37°C) and isoDD:IPA (37°C).***(d1 – d3)** Schematic representation of light scattering due to surface structuration corresponding to three different surface morphologies observed for CD wax films on quartz plates. (For interpretation of the references to colour in this figure legend, the reader is referred to the Web version of this article.)

Here, in order to establish understanding on structure induced optical reflectance, we focus on coating conditions that lead to significantly different surface morphologies as well as length scale of structures (Fig. 1).

Optical microscope images in Fig. 1 show the surface morphologies obtained following self-assembly of CD wax during evaporative drying from solution mixture of *isoDD:IPA* (90:10 v/v) at deposition temperature of 25 °C (Fig. 1: a1), 37 °C (Fig. 1: a2) and *isoHD:IPA* (90:10 v/v) at 37 °C (Fig. 1: a3). Images in Fig. 1 a1 and a2 clearly show that there is significant variation in surface structures on increasing deposition temperature from 25 °C to 37 °C. The surface of wax film is more homogenous and comprise of shallow structures on increase of deposition temperature. Whereas, with change in non-polar component of the solvent mixtures from isododecane *isoDD* to isohexadecane *isoHD* (from Fig. 1 a2 and a3 respectively), we observe similar shallow morphology. With scanning electron microscopy, we could visualize the shape of the wax crystals and the type of surface morphology. Further, from optical profilometry, we quantitatively define surface roughness in terms of average feature height (**h_F-avg_**) root-mean-square gradient (**SDQ**) and auto-correlational length (**SAL**). The qualitative implications of these parameters are mentioned in Table S2 of ESI. While for film from *isoDD:IPA* (90:10 v/v) at 25 °C shows mixed morphologies with globule-like as well as plate-like structures, films obtained from *isoDD:IPA* and *isoHD:IPA* at 37 °C show similar morphologies with wax crystals in the shape of plates which are oriented vertically and horizontally. From AFM imaging shown in Fig. S4 of ESI, we also understand that there is no additional structuration at lower length scale, hence, measurements from optical profilometery were considered for quantification of roughness parameters. The roughness parameters are detailed in Table 1.

**Table 1:** Surface morphology and roughness parameters of CD wax films obtained from three different solvent mixtures.

Solvent mixture (v: v)	T (°C)	Ra	Type of morphology	Roughness parameters		
				h_F_ (μm)	SDQ	SAL
Isododecane: IPA is*oDD:IPA* (90:10)	25	2.5	Globules + plates	–	–	–
Isododecane: IPA *isoDD:IPA* (90:10)	37	2.5	plates	6.8 ± 0.8	1.8 ± 0.3	3.5 ± 0.40
Isohexadecane: IPA *isoHD:IPA* (90:10)	37	2.4	plates	10.5 ± 4.0	2.2 ± 0.2	3.5 ± 0.96

Fig. 1: b shows the total %reflectance (**%R**) on irradiation with light in UV and visible range. None of the structured films preferentially reflect visible light throughout the spectra as **%R** nearly remains constant. This is clear distinction from structural colour, which shows reflectance peak due to constructive interference of light waves reflected by the nanostructures array [14]. Among the coated CD wax films, films from *isoDD:IPA* (25 °C) and *isoHD:IPA* (37 °C) show significantly higher **%R**, with average reflectance of 35 %. In contrast, film from *isoDD:IPA* at 37 °C shows high transmittivity and poor reflectivity (comparable to that of **%R** of quartz plates) along the whole spectra. Interestingly, in UV range, all films show monotonic decrease in **%R** as well as **%T** evidently implying inherent absorption, which can be attributed to chemical heterogeneity of CD wax. This could also be verified from UV absorption spectrum of CD wax in solution state (Fig. S5) where the absorption bands lie at ∼ 208 and 330 nm. In such cases, the optical response throughout the spectra is contributed both by the chemical composition of the wax as well as the surface structures. Consequently, the sole influence of physical structuration is difficult to deconvolute. However, visibly films with high **%R** exhibit whiteness whereas film with shallow structures is transparent (95% in transmittance). Whiteness of the films could be attributed to higher **%R** which results due to multiple scattering at air-film interfaces (shown in Fig. 1: scheme d1) created by the 3D structures (scattering elements) causing incident light to scatter randomly in different direction from different planes leading to reduced transmission and higher total reflectance. Contrastingly, uniform and shallow structures for film obtained from *isoDD:IPA* (deposition temperature: ∼37 °C) effectively create very thin layer of air-film interface (as shown in Fig. 1: scheme d2) leading to poor scattering despite showing similar morphology to film obtained from *isoHD:IPA* (deposition temperature: ∼37 °C). As evident from the microscope images, all the surface morphologies obtained with CD wax are not periodic with characteristic length-scale resulting in lack of peak reflectance due to constructive interference [14]. Hence, the films appear white and devoid of any characteristic structural color.

To circumvent the contribution of material absorption on the optical response of the structured wax films, we explored self-assembly of *Myristyl Palmitate* (MP) wax on quartz plates. MP wax is chemically homogenous wax comprising of C_30_ and C_32_ esters, only, with zero absorptivity in UV region. This was also corroborated by absorption curve, shown in Fig. S5: b, that was obtained for MP wax in solution state. DSC measurements shown in Fig. S1 of ESI reveals low melting temperature of MP wax, unlike CD wax, with start of melting and melting point at ∼35 °C and ∼48 °C respectively. Due to low melting temperature, at higher deposition temperature, surface structuration would be governed by re-crystallization of wax from its melt rather than self-assembly by evaporative drying of solution. Therefore, change in deposition temperature was not considered for tuning surface structuration as before.

Three solvents/solvent mixtures: *IPA:CH* (50:50 v/v), *tPA* and *tPA:CH* (50: 50 v/v) were identified based on vapor pressure and their degree of solubility with wax. Degree of solubility of solvent mixtures with wax is determined by Hansen Solubility Parameter Distance (HSP distance, **Ra**) which considers each component of solubility parameter (**δ**) and higher **Ra** implicates poor solubility of wax in the solvent mixture. **Ra** for solvent mixtures were determined by running simulation with *HSPiP* software. Relevant solvent properties of pure mixture of solvents are mentioned in Tables S3 and S4 of ESI.

**Fig. 2:**
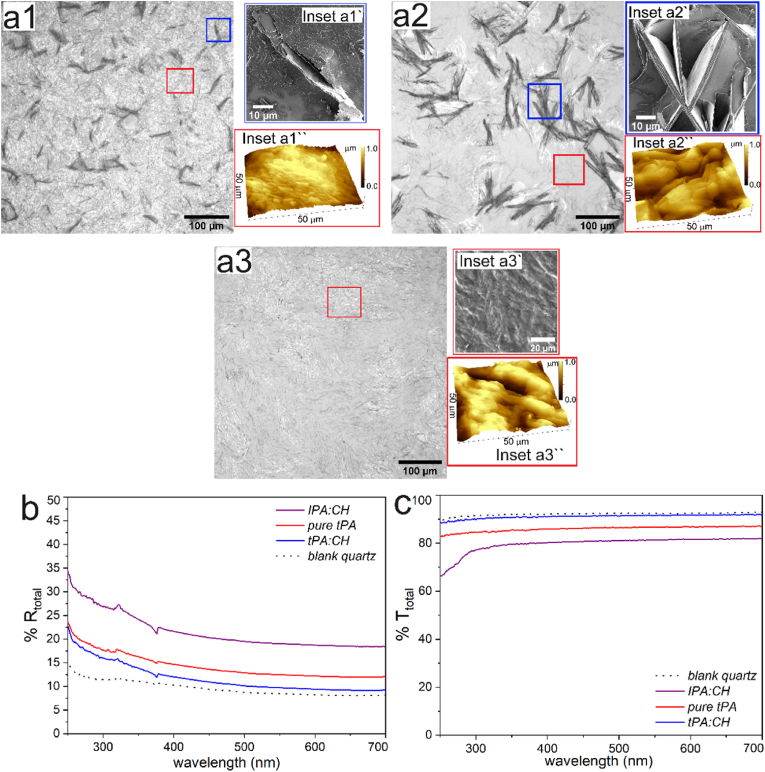
Surface morphologies obtained from coating of MP wax from its solution in (**a1 – a3**) *IPA:CH* (50:50 v/v); *tPA*; *tPA:CH* (50:50 v/v) respectively. Inset images showing corresponding surface morphology of structured MP wax films as imaged by (**a1′; a2′**) Scanning Electron Microscope and (**a1′′ - a3′′**) Atomic Force Microscope (50 × 50 μm^2^). *Scale bar in a1 - a3, a1′anda′2, a3′ represent 100, 10 and* 20 μm *respectively.* Total (**b**) reflectance (**%R_total_**); (**c**) transmittance (**%T_total_**) of structured MP wax films and blank quartz plate as measured by UV–vis Spectrophotometer.

Optical microscope images in series a of Fig. 2 reveal large area surface morphologies of as-coated MP wax on quartz plates. We observe two broad types of morphologies where films deposited from *IPA:CH* and *tPA* (Fig. 2: a1 and a2 respectively) are similar and comprise of micron scaled needle-like structures. From SEM images, we understand that the needle-like structures are formed due to deposition and aggregation of wax crystals in the form of plates which are aligned vertically. These vertically aligned plates contribute to 3D surface structuration of MP wax film. Interestingly, AFM surface imaging of flat zones (within red box) reveals additional roughness at lower length scale which is evident in inset images of Fig. 2 a1′′ - a3′′. These arise due to sequential stacking of wax plates in horizontal direction during self-assembly. Thus, re-crystallization of MP wax during evaporative drying from its solution leads to dual-scale morphology comprising of structuration at both micron and sub-micron length scale. Contrastingly, OM image in Fig. 2: a3 show no such needle-like structures confirming single-scale morphology with roughness at sub-micron length scale due to stacking of horizontally oriented wax plates only for film from *tPA:CH*. To precisely quantify the roughness of films showing dual scale morphology, we considered two **SDQ** values: **SDQ_1_** for a large area with needle-like structures (represented with blue square box in OM images of Fig. 2 a1 and a2) and **SDQ_2_** of an area with horizontally stacked plates (flatter zones represented within red box of each optical microscope image in series a of Fig. 2). The values of the roughness parameters for the above-mentioned MP wax films are provided in Table 2.

**Table 2:** Surface morphology and roughness parameters for films deposited from MP wax solution in three different solvent/solvent mixtures on quartz plates

Solvent/Solvent mixture	Ra	Type of morphology	Roughness parameters			
			h_F_ (μm)	SAL (μm)	SDQ_1_	SDQ_2_
*IPA:CH*	6.8	Dual	3.1 ± 1.2	2.65 ± 1.2	0.95 ± 0.02	0.99 ± 0.05
*tPA*	8.5	Dual	12.1 ± 2.5	24.5 ± 5.5	2.65 ± 0.04	0.55 ± 0.13
*tPA:CH*	3.1	Single	–	–	–	0.88 ± 0.10

∗Film thickness for all films is in the range of 0.8–1.0 μm.

As discussed in our previous work [44], during evaporation of solvents, the interplay of evaporation kinetics and re-crystallization dynamics is critical in driving physical structuration on wax films. Since, MP wax comprises of only esters, wax crystal re-crystallizes in the form of plates. However, the orientation of the wax plates is governed by **Ra** and evaporation kinetics of the solvents. For *IPA:CH* and *tPA*, although starting **Ra** is closer (Fig. S6 of ESI), re-crystallization dynamics gradually becomes faster for *IPA:CH* in comparison to *tPA* due to its progressive increase in **Ra** with rapid evaporation of isopropanol and cyclohexane in the solvent mixture. Moreover, slower evaporation kinetics of *tPA* compared to *IPA:CH* allows adequate time for wax crystals to grow and form aggregates during gradual evaporation. This leads to aggregation, which is manifested as larger wax plates, both laterally and vertically, resulting in higher **h_F-avg_** and **SAL** for film obtained from *tPA* in comparison to *IPA:CH* film. In contrast, we obtain smoother film, devoid of any micron-scaled 3D structuration, for film from *tPA:CH*. This can be attributed to considerably lower **Ra** (better solubility than *IPA:CH* and *tPA*) and faster evaporation kinetics resulting in late re-crystallization and lesser duration for crystal (plate) growth leading to smaller wax plates which orients in horizontal direction without forming any aggregates.

Fig. 2: b and c show the optical response of structured MP wax films as measured by UV–vis Spectrophotometer with integrating sphere. From the spectral curve, similar to CD films, we observe that structured MP films do not preferentially reflect light along the whole spectra. **%R_total_** is approximately constant throughout the visible spectra which visually gives white appearance similar to CD films. However, unlike CD films, there is a monotonic increase in **%R** from UV-A to UV-C (Fig. 2: b and Table 3). Of all the structured MP wax films, dual-scale morphology shows higher **%R_total_** (red and purple curves) throughout the spectra, while there is an increase of **%R**∼ 4–5 % with MP wax comprising single scale morphology. Several studies have reported that enhanced scattering is due to physical structuration of epicuticular wax on plant leaves where shape, orientation and density of the crystal structures govern the spectral shape and wavelength-dependent reflectance [11,[48], [49], [50]]. Since, MP wax has zero inherent absorption throughout the spectra (250–700 nm) and thickness of all films are similar at 0.9 ± 0.1 μm (details of film thickness in section S4 of ESI), increase in **%R_total_** (compared to uncoated quartz) can be attributed to presence of physical structuration due to self-assembled crystals. The homogenous nature of MP wax leads to crystals only in the shape of plates on film formation for all films. Therefore, shape of scattering object/crystals has no impact on observed differences in optical response between the films. However, orientation of the plates varies for dual and single scale morphology. Typically, higher reflectance in UV can occur either due to offset of **%R** along the visible spectra or by preferential scattering by structures [47]. We quantify the steepness or the rise in **%R** of UV spectra relative to visible spectra by ratio of **%R**_**|λ = 350nm**_**/%R**_**|λ = 600nm**_ (noted by **r** in Table 3). Despite minimum **%R_total_** close to uncoated quartz, single scale morphology (or horizontally stacked plates) obtained from *tPA:CH* shows maximum increase in UV scattering with value of **r** at 1.45 (in Table 3). Contrastingly, **r** for *IPA:CH* film is ∼1.30 and higher absolute **%R** value in comparison to *tPA* and *tPA:CH* films in the UV spectra. This is due to the observed offset in visible spectra which results from enhanced scattering by the micron scaled vertically oriented plates, absence of which decreases **%R_total_** as evident from total reflectance of *tPA:CH* film (blue solid line). This comparison reveals contribution of plate orientation on scattering properties where the micron-scaled 3D structures enhance scattering in visible spectra while sub-micron scale roughness is significant in UV spectra.

**Table 3:** Optical properties of structured wax films obtained from solution of MP wax on quartz plates.

Solvent	Morphology	Total reflectance (%R)			r = %R_|λ = 350nm_/%R_|λ = 600nm_
		UV-C (250–280 nm)	UV-B (280–315 nm)	UV-A (315–400 nm)	
*IPA:CH* (50:50)	Dual	30	25	22	1.30
*tPA*	Dual	22	21	17	1.33
*tPA:CH* (50: 50)	Single	20	17	14	1.45

Unlike CD films for which surface features are disordered and dense, MP wax films show long-range ordering where the micron-sized wax crystals are randomly oriented but periodically spaced from each other which is quantified by the value of **SAL** obtained from optical profilometer measurements. Between films obtained from *IPA:CH* and *tPA*, we observe a reflectance offset of ∼7 % with similar value of **r** at 1.30. We believe that the density of crystal plates (represented by **SAL)** is higher (**SAL** for ***tPA∼*** 24.5 μm vs **SAL** for *IPA:CH* ∼ 3 μm) and contributes to this difference in reflectance. Higher **SAL** indicates larger area between vertically oriented plates increasing the area of flatter zones which are zones with sub-micron scale roughness (represented by **SDQ_2_** in Table 3). **SDQ_2_** for *tPA* is lower than both *IPA:CH* and *tPA:CH* films resulting in less scattering and manifested as lower **r** as well as insignificant increase **%R_total_** in visible spectra. Consequently, fraction of transmitting radiation increases which is reflected in higher values of **%T_total_**. These results show the importance of density of scattering objects for efficient optical scattering, where vertically oriented plates should be at optimal periodicity and not too far from each other (*i.e.,* leading to high value of **SAL**), which was also corroborated in a report by *Luke* et al. [47] Accordingly, we observe no significant scattering in visible spectra for *tPA:CH* film due to absence of vertically oriented plates.

Our experiments on model quartz substrates with MP wax highlights the influence of structuration and its length scale on the optical performance of the coating. The spectral shape and maximum **%R_total_** achieved in UV domain is 30–40 % which is at par with reported reflectance by *Middleton* et al. [14] for specific species of blueberries that bear self-assembled slab-like (resembling plate-like) wax structures. Increased photo-protection is manifested as reduced transmissivity of radiation through the substrate which can be achieved either by increased scattering or by increasing the film thickness. Scattering effect is observed for film thickness greater than transport length (distance travelled without scattering) which also depends on refractive index of the material [48]. In this case, with series of controlled experiments (discussed in section S10 of ESI) we understand that film thickness as high as ∼5 μm is less than transport length and not enough to drastically reduce **%T**. For similarly thick film or less, this can substantially be improved by surface structuration which attains maximum for dual scale morphology at an optimum length scale as discussed above.

### 3.2 Replication of structuration on model skin surfaces: PMMA plates

Here, we adopt similar approach for structuration of realistic substrates of cosmetic interest where we focus on replication of structures with maximum optical performance on PMMA plates. Due to the close resemblance with human skin from the standpoint of wettability and intrinsic roughness, PMMA plates are commonly utilized as model substrates for photo-protection evaluation in cosmetic applications [51]. Before deposition of MP wax solution, inherent nature of the substrates is an important factor that needs to be considered. PMMA plates (∼40 mJ/m^2^) have lower surface energies in comparison to quartz plates and show roughness of the order of few tens of microns that are supposed to mimic the roughness of human skin. The surface characteristics of these substrates are detailed in section S2 of ESI. To achieve complete surface coverage on textured substrates, we tuned coating conditions accordingly to circumvent dewetting of deposited solution layer induced by substrate roughness and non-wettability that typically leads to incomplete coverage. Therefore, **Ra**, vapor pressure and concentration of wax in solution (***C*_*n*_***)* were tuned by varying solvents (solvent mixtures) used for solubilization of MP wax. Additionally, for PMMA plates, we avoided coating of wax from polar solvents as it significantly solubilizes the substrate surfaces due to high substrate – solvent interactions.

**Fig. 3:**
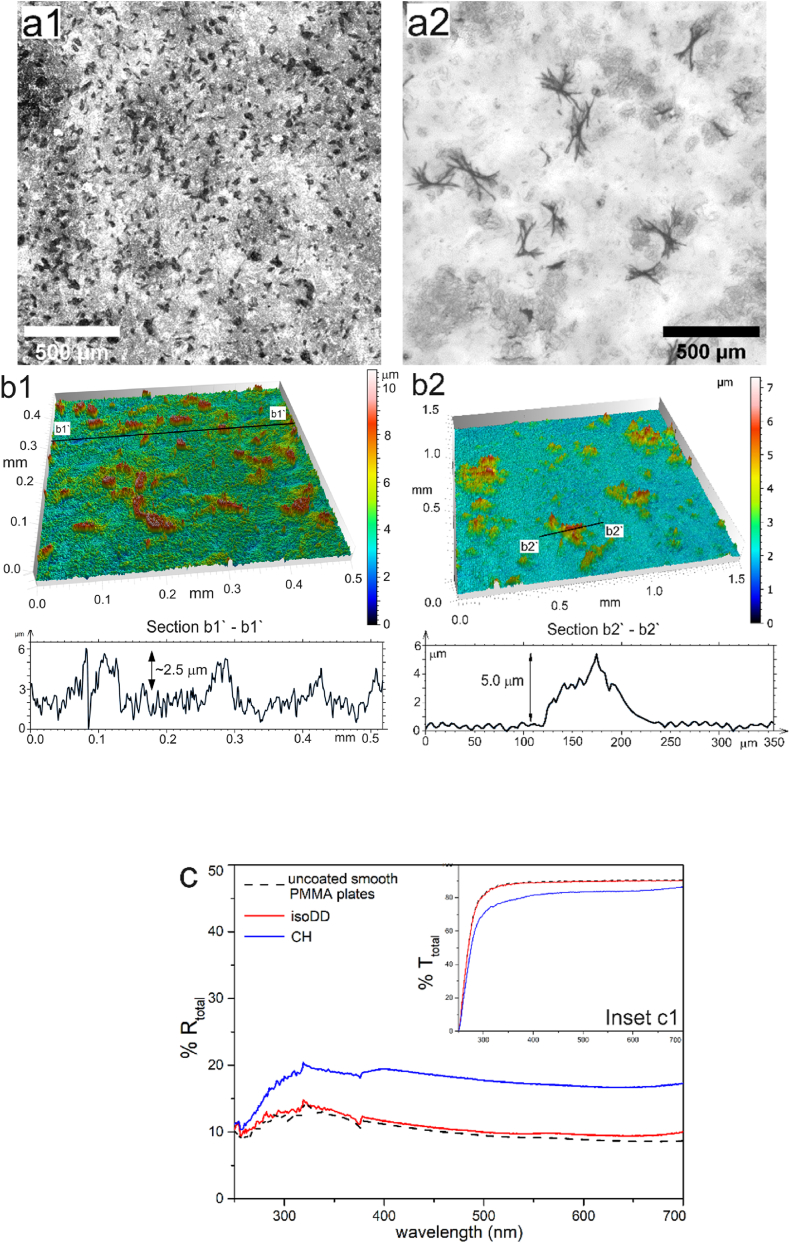
Optical microscope images of MP wax films obtained from solution in (**a1**) *CH*; (**a2**) *isoDD*. *Scale bar in each image represents* 500 *μ*m. (**b1**, **b2**) Corresponding 3D representation of the surface of MP wax films as scanned by optical profilometer. Section b1 – b1 and b2 – b2 represent the line profile of the wax films. (**c**) Total reflectance (**%R_total_**) as measured by UV–vis spectrophotometer for MP films on PMMA plates from *isoDD* (red solid curve) and *CH* (blue solid curve). Corresponding total transmittance (**%T_total_**) of the films shown in (**inset c1**). *All films were deposited from wax solution at a concentration of****C*_*n*_***∼10.0 % (w/v). (For interpretation of the references to colour in this figure legend, the reader is referred to the Web version of this article.)*

Optical microscope images in series a of Fig. 3 show similar dual-scale morphology as wax films on quartz plates. With change in solvent, particularly the vapor pressure, it is possible to obtain similar morphology as films on quartz plates, however, length scale of the structures varies on PMMA substrates. The roughness parameters for the films deposited from different solvents are detailed in Table 4. Like previous case, here, we obtain 3D structuration due to deposition of wax plates in the vertical direction which can be visualized as needle-like structures from optical microscope images.

**Table 4:** Surface morphology and roughness parameters for films deposited from corresponding MP wax solutions on PMMA substrate and SC.

Substrate	Solvent	Type of morphology	Roughness parameters			
			h_F_ (μm)	SAL (μm)	SDQ_1_	SDQ_2_
Smooth PMMA	*isoDD*	Dual	5.3 ± 1.2	55 ± 12	0.44 ± 0.19	0.15 ± 0.07
Smooth PMMA	*CH*	Dual	2.5 ± 0.8	15 ± 7	0.42 ± 0.15	0.40 ± 0.06
SC	*isoDD*	Dual	35 ± 5.2	180 ± 15	1.50 ± 0.05	0.68 ± 0.05
SC	*CH*	Dual	4.0 ± 1.1	41.1 ± 10.5	0.58 ± 0.02	0.55 ± 0.05
SC	*IPA:CH*	Dual	12.0 ± 3.5	47.2 ± 2.3	1.30 ± 0.06	0.85 ± 0.02

With optical profilometry, the average feature height (**h_F-avg_**) could be determined which is ∼2.5 μm for *CH* films and ∼5.5 μm for *isoDD* film. Although both films (from *CH* and *isoDD*) show similar dual-scale morphology, for *CH* film, **SAL** ∼ 18 μm is lower than to *isoDD* film (**SAL** ∼ 55 μm) implying that 3D structures due to vertically aligned wax plates are denser and closely spaced for *CH* film in comparison to *isoDD* film. The **SDQ_1_** representing sharpness of structures for large areas consisting of micron as well as sub-micron scaled structures is comparable for *isoDD* film and *CH* film, however, **SDQ_2_** (for flatter zones) is higher for *CH* film than *isoDD* film. The optical response of MP wax films on PMMA substrate as shown in Fig. 3: c show higher **%R** (∼20 %) for *CH* film in comparison to *isoDD* film. **%R** for *isoDD* film is similar to that of underlying PMMA substrate which proves that the length scale of the structures cannot efficiently scatter incident light albeit with the dual-scale morphology. Particularly, lower **SDQ_2_** and higher **SAL** values represents larger smoother or flatter zones which contributes to decreased **%R** and thereby increase in **%T** (Fig. 3: inset c1). This is in parity with optical response observed for films on quartz substrate, where dual-scale morphology (shown in Fig. 2: a2) with lower **SDQ_2_** and higher **SAL** show lower **%R** and higher **SDQ_2_** leads to enhancement in **%R**. Additionally, significant decrease in **%T** for both films on PMMA substrates from 315 nm onwards is likely a manifestation of absorption of PMMA which is known to have high absorption coefficient in far UV region [52]. In this case, due to the substrate absorption in UV-B region, we do not quantify and compare the values of **r**. Nonetheless, correlation of higher **%R_total_** (scattering effect) with feature length scale of the structures comply with dependencies observed for wax coated quartz plates.

**Fig. 4:**
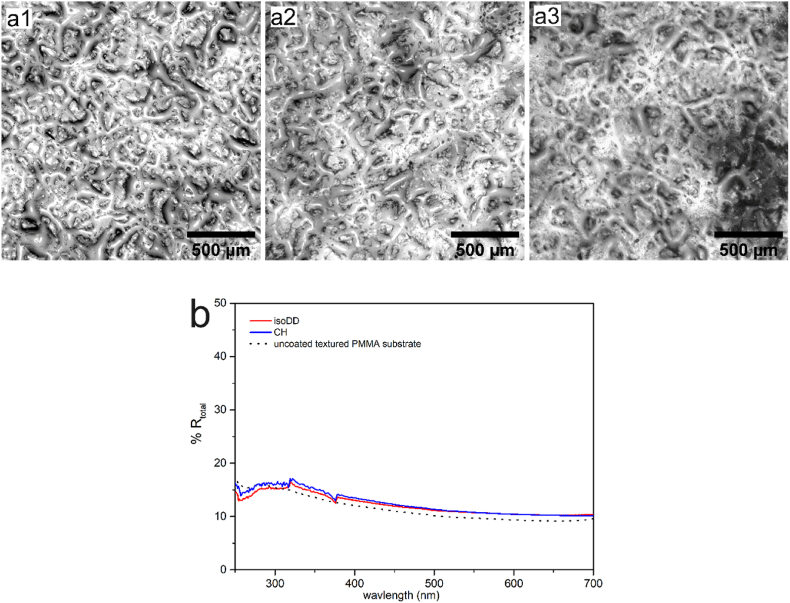
Optical microscope images of (**a1**) uncoated textured PMMA substrate and coated with wax films from solution of MP in (**a2**) *isoDD*; (**a3**) *CH*. All scale bars represent 500 μm. (**b**) Total reflectance (**%R**) as measured by UV–vis spectrophotometer for MP films on PMMA plates from *isoDD* (red solid curve) and *CH* (blue solid curve). (For interpretation of the references to colour in this figure legend, the reader is referred to the Web version of this article.)

Interestingly, UV–vis measurements show no enhancement in **%R** for coating from same wax solution on textured PMMA substrates (Fig. 4). Intrinsic roughness of PMMA substrate is detailed in section S2 of ESI, which have *wave-like* patterns with average height of ∼30 μm.

Presence of "*wave-like"* patterns on PMMA plates essentially disrupt formation of needle-like 3D structuration and additional roughness to the textured PMMA plates. Consequently, optical performance remains similar for coated as well uncoated PMMA plates showing no enhanced light scattering due to structuration of the PMMA surface. To obtain uniform coating and structuration, coating a thicker wax film by increasing concentration of wax in the solution (***C*_*n*_**) is a feasible option. In this regard, we would like to emphasize that increasing ***C_n_*** decreases the stability of wax in the solution leading to phase separation of wax from the solvent immediately which ultimately leads to uneven deposition. Moreover, a thicker film would override the existing patterns or roughness diminishing its influence on light scattering. Consequently, within the cosmeceutical limits of the coating conditions, we do not observe distinct dual scale morphology similar to the ones observed on quartz and smooth PMMA plates.

### 3.3 *Invitro* replication of structuration on real skin surface: *StratumCorneum*

As a biomimetic approach for UV photo-protection, we explored patterning real skin surfaces *in vitro*, *i.e., Stratum Corneum* (SC) with MP wax. Being the uppermost dermal layer, SC has natural tension lines which are in the form micro-structures (microreliefs) with an average height ∼ 25 μm and surface energy of ∼25 mJ/m^2^ [53]. The roughness parameters and substrate characterization are detailed in section S2 of ESI. As mentioned earlier, these microreliefs are imperative for sensorial and tactile perception as it links to the mechanoreceptors in the underlying dermal layers [41], and are responsible for sensorial reactions to touch, temperature and other stimuli.

**Fig. 5:**
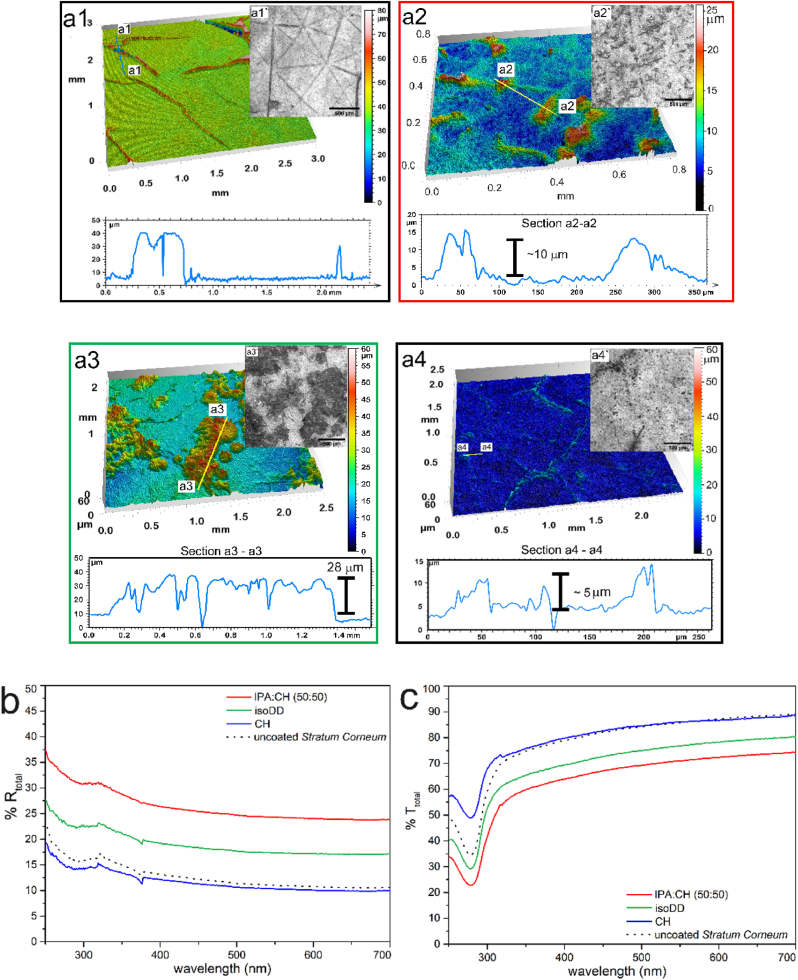
3D representation of structured MP wax film on SC deposited from (**a1**) bare (uncoated) SC; (**a2**) *IPA: CH* (50:50); (**a3**) *isoDD* and (**a4**) *CH* as obtained by optical profilometry. Total (**b**) reflectance (**%R**) (**c**) transmittance (**%T**) of structured MP wax films and blank quartz plate as measured by UV–vis spectrophotometer.

Here, by adopting similar approach as before, we focus to superpose new structures on existing ones with coating of MP wax to retain the skin's tribological properties as well as enhance structure induced reflectance of the surface. Therefore, we accordingly tune the coating conditions to retain skin's topography due to microrelief as well as obtain structuration from self-assembly of wax. We identified three different solvent mixtures for coating of wax solutions that lead to such structuration, as shown in optical microscope images in Fig. 5.

Comparison of optical microscope images of bare or uncoated SC (Fig. 5: a1) with wax coated SC (Fig. 5: series a2 – a4) reveal structuration due to deposition of waxes, which is governed by re-crystallization of wax from its solution in *IPA:CH* (Fig. 5: a1), *isoDD* (Fig. 5: a2) and *tPA:CH* (Fig. 5: a3). All three films show dual-scale morphology which could be verified by surface imaging with optical profiler and atomic force microscope (Fig. S9 of ESI). Here, it is worth noting that scale bar for all structured wax films on SC represents 0–50 μm for visual comparison of the surface roughness and therefore, the structures for flatter film are at lower scale bar. From quantification of roughness parameters with optical profilometer measurements, we understand that length scale of the structures is dependent on the casting solvents/solvent mixtures. The roughness parameters as determined are detailed in Table 4. Here, SCs show discrete and sharp micro-structures (Fig. S2: c) in contrast to "*wave-like*" patterns on textured PMMA plates. This allows uniform structuration of the flatter areas on SC, unlike PMMA plates where the curvature of the patterns hinders vertical orientation of wax plates (leading to 3D patterns) resulting in shallow structures. As a result, despite the intrinsic roughness of similar length scale on both SC and PMMA plates, we observe formation of dual scale morphology with MP wax on SC only. Qualitatively, this highlights the nature of the substrate textures that lead to desirable structuration in this case.

Film from *IPA:CH* with intermediate **h_F-avg_** ∼ 12 μm and **SDQ_2_** ∼ 0.85 show maximum reflectance with average **%R** (red solid line) in UV spectra of 32 %. The average %R for all structured wax films on SC in each UV spectra is detailed in Table 5. Despite showing dual-scale morphology, poor scattering of structures on films from *isoDD* and *tPA:CH* can be attributed to the roughness parameters. Although, film from *isoDD* show large aggregates with **h_F-avg_** ∼35 μm, the aggregation is too scattered with **SAL** ∼185 μm thereby increasing the flatter zones with **SDQ_2_** ∼ 0.65 resulting in decreased **%R**, despite similar **SDQ_2_** for *IPA:CH* and *isoDD* films. This follows a similar trend as observed for reflectance of wax films on quartz and PMMA substrates, where dual-scale morphology with optimal **SAL** and higher **SDQ_2_** show maximum scattering throughout the spectra. On the other hand, from Fig. 5: a3, we observe similar vertically oriented crystal plates for *CH* film with **h_F-avg_** and **SAL** of ∼3 μm and ∼40 μm, respectively. However, optical measurements in Fig. 5: b show similar **%R** to bare SC (blue vs black dotted curves) which could be attributed to shallow nature of the structures with respect to microreliefs of SC (**h_F-avg_** ∼ 25 μm). Consequently, these vertically oriented wax plates of lesser **h_F-avg_** fails to act as additional scattering elements besides the microreliefs of SC. Unlike films on quartz plates and PMMA substrate, here, total reflectance or scattering is also dependent on the feature height of the wax structures (**h_F-avg_**) which, based on our experiments, should at least be of the same order as the microreliefs for crystal structures to induce additional scattering.

**Table 5:** Optical properties of structured wax films obtained from solution of MP wax on SC.

Solvent	Morphology	Total reflectance (%R)		
		UV-C (250–280 nm)	UV-B (280–315 nm)	UV-A (315–400 nm)
*IPA:CH* (50:50)	Dual	30	25	22
*tPA* (pure)	Dual	22	21	17
*isoDD* (pure)	Single	20	17	14

With this approach, we successfully pattern real *in* *vitro* skin surfaces which improves UV scattering ability and also retains inherent skin's topography. Our experiments show that light scattering is maximum for structures with feature height at par or more than the existing microreliefs. To obtain desired structural length scale, it is crucial to optimize the coating conditions by tuning parameters like **Ra** (by choice of solvent with respect to wax) and ***C*_*n*_** (concentration of wax in the solution).

### 3.4 Wettability of biomimetic epicuticular wax films

**Fig. 6:**
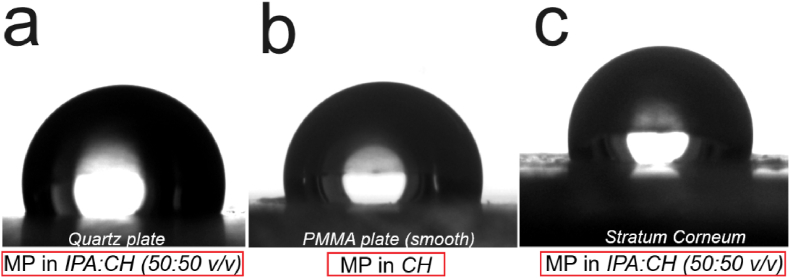
Water contact angle of optically best performing (maximum **%R_total_**) structured MP wax films. Films obtained from its solution in (**a**) *IPA: CH* (50:50 v/v) on quartz plate; (**b**) *CH* on PMMA plate (smooth); and (**c**) *IPA: CH* (50:50 v/v) on SC.

We presented biomimetic approach to texturize skin surfaces *in* *vitro* with preceding experiments, however durability of the waxes on the skin surfaces is another challenge that needs thorough investigations from the standpoint of cosmetic applications. As preliminary investigation in this regard, we studied the wettability of the wax coated substrates. To determine the wettability of films, we measured water contact angle on wax coated substrates that showed maximum optical reflectance. Fig. 6 shows the water contact angle on structured wax films showing maximum UV-reflectance on quartz, PMMA plate and SC, respectively.

Irrespective of the type of morphology or the length scale of the structures, WCA is similar on all the films at 105 ± 2°, implying no significant influence of structuration on wettability of the films. This could be attributed to the fact that none of the structures obtained with *Candelilla* and *Myristyle Palmitate* satisfy the structural length-scale required to achieve Cassie-Baxter state of wetting which fundamentally models structure induced super-hydrophobicity [9]. However, we observe that all the films are hydrophobic with water contact angle ∼ 105–110° (Fig. 6 and Table S6). This highlights that while these surface structures mimics plant-based optical reflectivity, simultaneously it also displays hydrophobicity, which is often a desired property for cosmetic products.

## 4 Conclusions

Following evaporative drying induced self-assembly of *Candelilla* and *Myristyl Palmitate* plant-based waxes, we obtain diversely structured films at wider length scale with specific epicuticular wax on quartz plates by tuning the coating conditions and solvent properties. With this work, we reveal the optical properties, in details, of such structured biomimetic films by measuring total reflectance (**%R**) and transmittance (**%T**) using UV–vis Spectrophotometer and highlight the contribution of physical structuration on UV-reflectance. While it is evident from optical measurements that *Candelilla* films with mixed surface morphology, comprising of globules and plates, scatter light efficiently in visible spectra, it is rather difficult to deconvolute and relate reflectance with physical structuration due to the inherent UV absorption by *Candelilla* wax.

To overcome the influence of material absorptivity on the optical response, we replicate similar surface structuration with *Myristyl Palmitate*, which is chemically homogenous and non-absorbing wax, in UV range especially. As a result, we could directly correlate surface morphology to reflectance due to its zero absorptivity in UV and visible range. By tuning solvent properties, we obtain broadly two types of surface morphologies which are: (i) *dual-scale* surface morphology comprising micron-scaled needle-like structures as well as roughness at sub-micron level, and (ii) *single-scale* morphology with roughness at sub-micron scale only. Vertically oriented wax crystals in the shape of plates result in micron-scaled 3D structures whereas horizontal stacking of these plates during deposition leads to smoother films with roughness at sub-micron scale. We understand that self-assembled 3D crystal structures act as scattering elements and improves reflectance particularly in visible spectra by 10–12 %. The importance of sub-micron scale roughness is rather highlighted by enhanced **%R** (of 5–7 %) preferentially in UV spectra for single-scale morphology. Therefore, total reflectance is enhanced for dual-scale morphology at optimum length scale which depends particularly on orientation and density of crystal plates. With this biomimetic approach, we also show that *in-situ* replication on skin of structures alone leads to absolute reflectance values in visible spectra ∼20 % and UV ∼20–35 % which is comparable to reported reflectance on plant leaves with slab-like crystal structures [14] and waxy *Eucalyptus* plant leaves [11].

To highlight the feasibility of this approach for skin care applications, we replicate best performing structures on PMMA plates (model skin surfaces) and *in* *vitro* on real skin surfaces, *i.e. Stratum Corneum* (SC). The optical response of structured MP films on *smooth PMMA plates* and SC reveal similar dependency on surface morphology and length scale of structures as quartz plates. Structuration of these substrates with MP wax enhances total reflectance by 15–20 % in UV spectra. Moreover, we establish relation between the length-scale of structures that efficiently scatter light and substrate roughness. However, with similar method of structuration, it is not possible to enhance reflectivity of the textured PMMA substrate as continuous "*wave-like*" patterns disrupt uniform deposition of wax crystals hindering superposition of additional structures leading to dual-scale morphology, despite showing similar length scale of substrate roughness as SC. This clearly highlights the importance of geometrical nature of substrate textures on obtaining desired structuration with waxes.

Herein, we particularly highlight the contribution of physical structuration on reflectance of light. From optical characterizations of several wax films, we identify the type of surface morphology and optimum length scale to achieve maximum reflectance of ∼32 % in UV spectra. This is in parity with reflective properties obtained from structuration of epicuticular wax on plant leaves naturally [11,14]. Since crystal structures depend on the chemical components of wax influencing surface structuration, similar phenomenon can be obtained from other waxes with different crystal structures (rod, ring-shaped), however, detailed studies in this regard are not within the scope of the present article. Further, we successfully show replication of wax structures *in-situ* on real and model skin surfaces highlighting the need to consider the nature and length scale of substrate roughness as microreliefs of skin are diverse which depend on parts of human body resulting variation in inherent roughness and physico-chemical properties. Overall, this study is a primary step towards development of bio-inpsired technique adopting physical defense method to enhance UV photo-protection and gradually rely less on chemical active ingredients.

In context of this work, we would like to emphasize that multiple functionalities of epicuticular wax arise due to intricate relation between number of factors besides surface structuration. The simultaneous *in-situ* replication of multitude of functionalities with specific physical structuration is challenging and requires tuning of length scale as per demand. Nevertheless, we present biomimetic films from plant based epicuticular wax showing both hydrophobic and UV photo-protective properties which have potential for dermatologic or cosmetic applications.

## CRediT authorship contribution statement

**Anuja Das:** Writing – review & editing, Writing – original draft, Visualization, Validation, Investigation, Formal analysis, Data curation, Conceptualization. **Luca Polacchi:** Writing – review & editing, Visualization, Validation, Project administration, Data curation, Conceptualization. **Jean-Yves Fouron:** Methodology. **Antoine Montaux-Lambert:** Methodology. **Laurent Billon:** Writing – review & editing, Supervision, Project administration, Methodology, Funding acquisition, Conceptualization. **Gustavo S. Luengo:** Writing – review & editing, Supervision, Methodology, Conceptualization.

## Funding

This work was funded by L'OREAL in the framework of the Self-Assembly of Waxes SAW project.

## Declaration of competing interest

G.S.L., L.P., J.-Y.F. and A.M.L are employees of L’OREAL engaged in research activities. The rest of authors received funding from L’Oreal.

## Data Availability

No data was used for the research described in the article.

## Glossary:

**H:**: Film thickness (μm)
**h_F-avg_:**: Feature height (μm)
**SDQ:**: Root-mean square gradient
**SAL:**: Auto-correlational length (μm)
**δ:**: Total Hansen Solubility Parameter (cal^1/2.^cm^−3/2^)
**δ_P_:**: Polar component of HSP (cal^1/2.^cm^−3/2^)
**δ_D_:**: Dispersive component of HSP (cal^1/2.^cm^−3/2^)
**δ_H_:**: Hydrogen-bonding component of HSP (cal^1/2.^cm^−3/2^).
