# Supplementary material for: Biomimetic UV photo-protection of skin surface by structured epicuticular wax films
Source: Mater Today Bio. 2025 Jun 17;33:101991. doi: 10.1016/j.mtbio.2025.101991 (PMC12226366; doi:10.1016/j.mtbio.2025.101991)
Supplement: Multimedia component 1 [file mmc1.docx]

**Electronic supporting information**

**Biomimetic UV photo-protection of skin surface by structured epicuticular wax films**

Anuja DAS^1^, Luca POLACCHI^2^, Jean-Yves FOURON^3^, Antoine MONTAUX-LAMBERT^2^, Laurent BILLON^1*^ and Gustavo S. LUENGO^2 *^

^1^ Bio-Inspired Materials Group : Functionalities & Self-Assembly, Université de Pau & Pays Adour, CNRS, IPREM UMR 5254, Technopole Hélioparc, 2 avenue Angot, 64053 PAU cedex 09, PAU, France.

^2^ L’Oréal Research & Innovation, 1 Av. Eugène Schueller, 93600 Aulnay-sous-Bois, Paris, France.

^3^L’Oréal Research & Innovation, 100 Av. de Stalingrad, 94550 Chevilly-Larue, France.

*To whom correspondence should be addressed: Laurent Billon ([laurent.billon@univ-pau.fr](mailto:laurent.billon@univ-pau.fr)) and Gustavo S. Luengo ([gluengo@rd.loreal.com](mailto:gluengo@rd.loreal.com))

**S1:** **DSC thermal analysis of *Candelilla* and *Myristyl Palmitate* wax as-acquired**

**
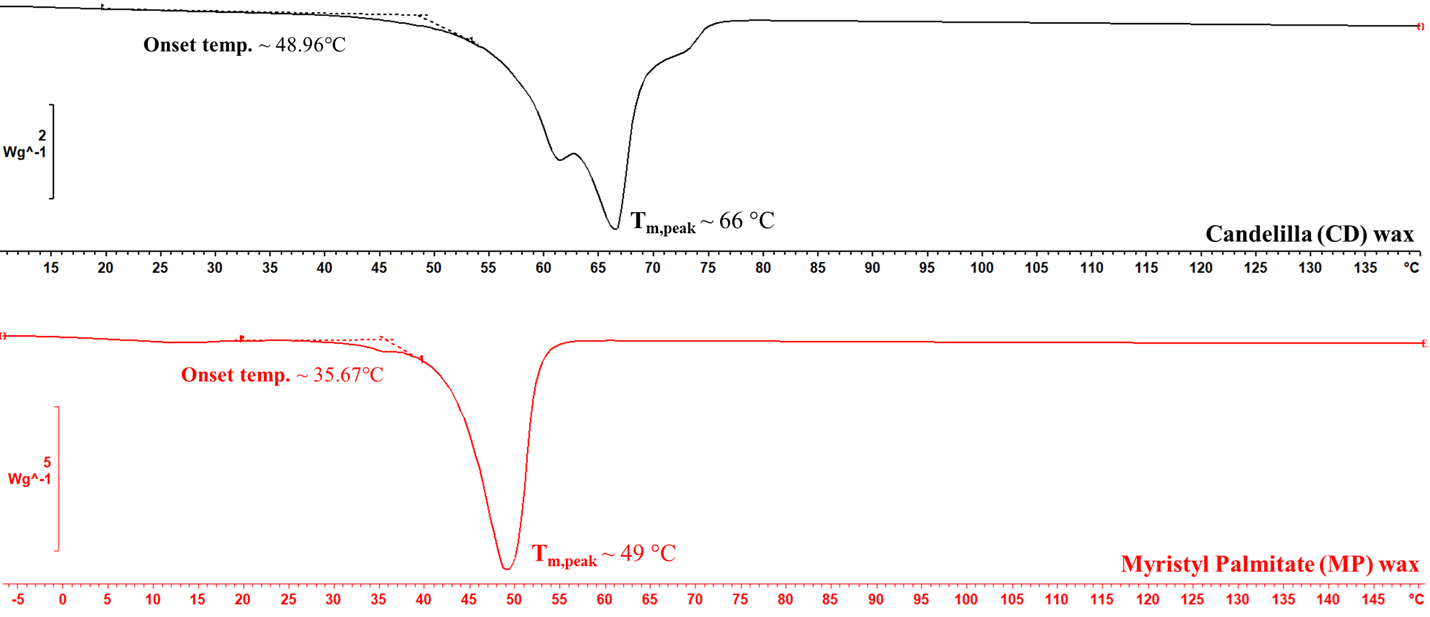
**

**Figure S1.** Heating curve recorded with DSC for (**a**) *Candelilla* (CD); (**b**) *Myristyl Palmitate* (MP) beads when heated to 150°C at 20°C/min. Onset of melting (**T_onset_**), melting peak (**T_peak_**) temperatures are as indicated in the curves.

**S2: Substrate surface properties**


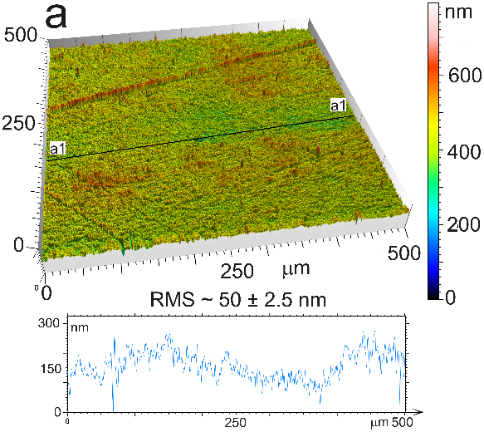
 **
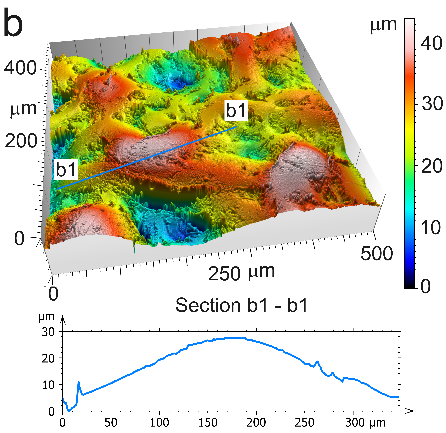
**


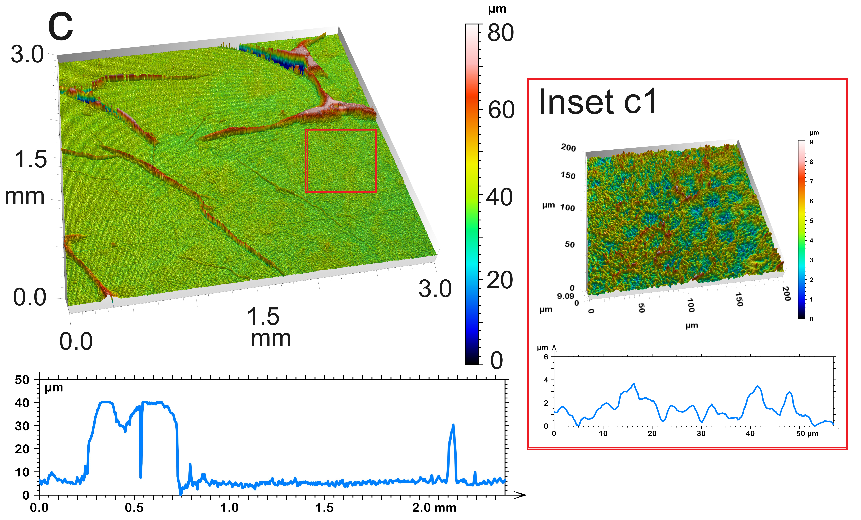
**
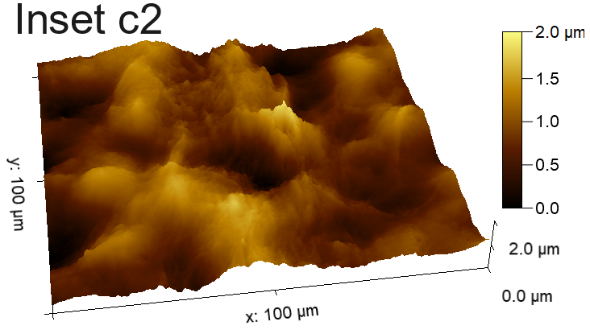
**

**Figure S2.** Surface roughness of *smooth* and *textured PMMA plates*, *Stratum Corneum* (SC) as measured by Optical Profilometry. 3D representation of (**a**) *smooth*; (**b**) *textured PMMA plates* and (**c**) SC. (**Inset c`**) area within the red box, from optical profilometer scans.

Two types of substrates were used for investigation as model skin-like surface. *PMMA plates* with smooth and textured surface. Optical profilometry of the surfaces are shown in Figure S2 a and b. The profile in the inset of each figure shows the variation in height across the line. The smooth plates have RMS ~ 50 nm while the textured plates have RMS ~ 8 µm. The texture on PMMA plates is in the form of peaks and valleys, the maximum height of which is approximately ~ 35 µm.

*Stratum Corneum* (SC) was used as skin-like surface comprising micro-reliefs in the form of folds with average height of each fold ~ 25 ± 5 µm. The apparently smoother area, marked in red rectangle box, has roughness at lower length scale with RMS ~ 1 µm

**Table S1.** Details of substrate roughness and patterns on PMMA plates and SC.

| **Substrate** | **Surface energy (mJ/m^2^)** | **Type of textures** | **Length scale of patterns & roughness** |
| --- | --- | --- | --- |
| *Stratum Corneum* | **~** 26[1] | Micro-structures (folds) | H (patterns) ~ 25 µm; 3.5 µm |
| PMMA smooth | ~ 40 | - | RMS ~ 50 nm |
| PMMA textured | ~ 40 | Peaks and valleys | H (patterns) ~ 35 µm, RMS ~ 7.5 µm |

**S3: Quantification of surface structuration**

Parameters used to quantify surface roughness for structured wax films.

**Table S2.** Roughness parameters for surface structuration on CD and MP (as-deposited) wax films.[2]

| **Roughness parameter** | | **Qualitative implication** |
| --- | --- | --- |
| **h_F-avg_** | Feature height | Average surface feature height |
| **SDQ** | Root-mean square gradient | Sharpness of the structures |
| **SAL** | Auto-correlational length | Density of structures |
| **RMS** | Root-mean-square-height | Average variation of feature height |

**S4: Film thickness of structured wax films by Optical Profilometry**

We measured film thickness for the wax film deposited at different conditions by optical profilometry. Since, defining film thickness for structured wax film is not straightforward, we considered thickness of the film as distance from substrate surface to an average line profile for air-film interface. This is schematically represented in Scheme S1 below.


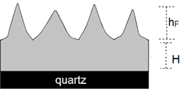


**Scheme S1.** Schematic representation of structured wax film with average feature height, **h_F_** and film thickness, **H**.

As measurement of thickness for such multi-component (unknown refractive index) rough film by ellipsometry is difficult, we performed scan with optical profilometer across a carefully made scratch on the films. This provides us with an approximate value of the film thickness, **H**. Figure S3 shows the line profile section across such scratch over two films *i.e.* drop casted CD (from *isoDD:IPA*) and spin coated MP (from *IPA:CH*) over quartz substrate.


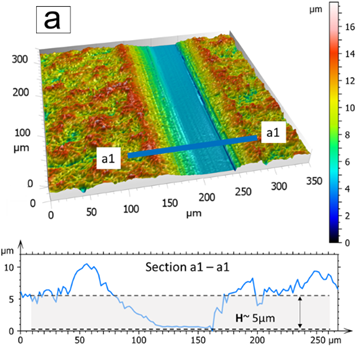

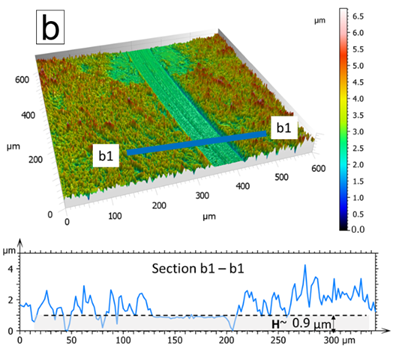


**Figure S3.** Optical profilometer images showing line profile across scratch on the structured wax film obtained by (**a**) drop casting *Candelilla* from its solution in *isoDD:IPA* at 37°C; (**b**) spin coating of MP from its solution in *IPA:CH* (50:50) at ambient condition. Line profile

As, final film thickness depends on coating or deposition conditions, we assume thicknesses to be similar for CD and MP films at specific coating conditions. For drop casting, the deposition volume was maintained at 625 µL (over 25 x 25 mm^2^ substrate area, **Cn**~1.0% w/v) to obtain a film with wax density of 1.0 mg/cm^2^, for all cases mentioned in the main manuscript. Spin coating parameters were fixed at speed = 500 r.p.m, time ~ 90s and volume = 250 µL. From profilometry, measured film thickness for drop casted wax film (Figure S3: a) is approximately ~5µm and spin coating (Figure S3: b) leads to film thickness of 0.8 – 1.0 µm .

**S5:** **Geometry of UV-vis Spectrophotometer with integrating sphere:**

.


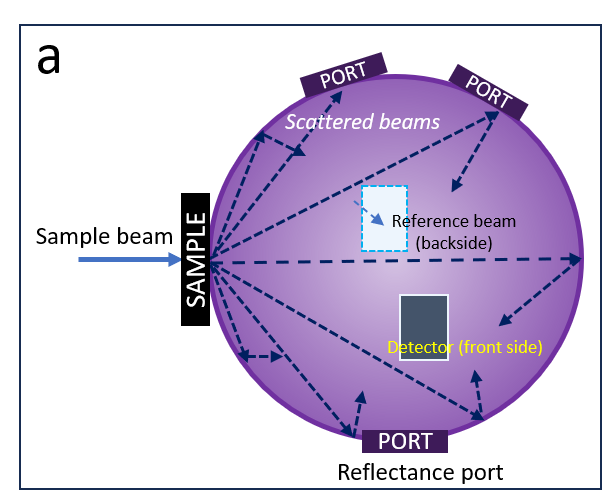

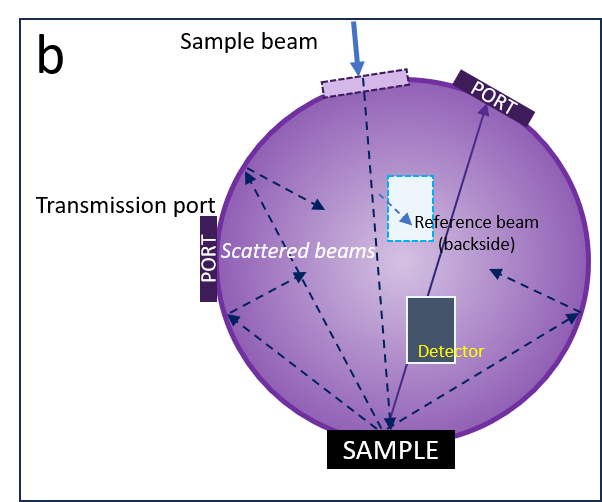


**Scheme S2.** Geometry of UV-Vis Spectrophotometer with 150 mm integrating sphere for measurement of optical properties of wax films in total (**a**) transmission; (**b**) reflectance mode.

**S6: Atomic Force Microscopy of *Candelilla* films**


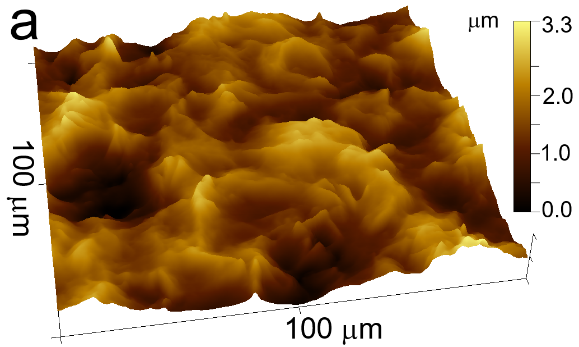

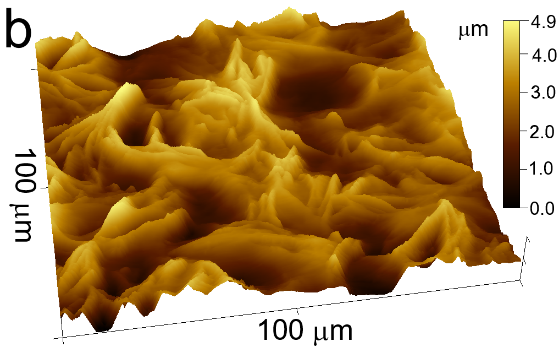


**Figure S4.** Surface morphology, as imaged by AFM, of structured CD films obtained from deposition of solution in (**a**) *isoDD:IPA* (90:10 v/v) (**b**) *isoHD:IPA* (90:10 v/v)

**S7:** **UV absorption spectrum for *Candelilla* and *Myristyl Palmitate* waxes**


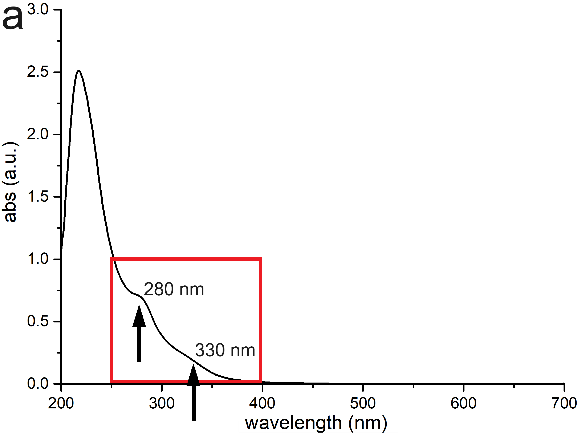

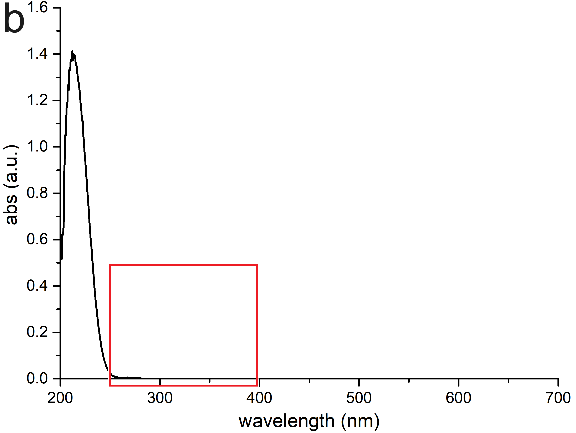


**Figure S5.** Absorbance curve for (**a**) CD; (**b**) MP solution in *isoDD:IPA* and *IPA* , respectively, showing absorption band of corresponding wax in UV-vis spectrum.

**S8:** **Solvent properties**

**Table S3.** Vapor pressure and components of solubility parameter of each solvent at 25°C and 35°C.

| **Solvent** | **Vapor pressure (mmHg)** | | **Solubility parameters (cal^1/2.^cm^-3/2^)** | |
| --- | --- | --- | --- | --- |
|  | **25°C** | **37°C** | **25°C** | **37°C** |
| *isoDD* | 1.53 | 3.32 | **δ_D_** = 14.8; **δ_P_** = 0.1; **δ_H_** = 0.1 | **δ_D_** = 14.5; **δ_P_** = 0.1; **δ_H_** = 0.1 |
| *IPA* | 81.50 | 154.00 | **δ_D_** = 15.5; **δ_P_** = 7.2; **δ_H_** = 12.8 | **δ_D_** = 15.1; **δ_P_** = 7.1; **δ_H_** = 13.9 |
| *isoHD* | 0.05 | 0.14 | **δ_D_** = 14.8; **δ_P_** = 0.1; **δ_H_** = 0.1 | **δ_D_** = 14.7; **δ_P_** = 0.1; **δ_H_** = 0.1 |
| *tPA* | 18.1 | 37.0 | **δ_D_** = 15.6; **δ_P_** = 5.0; **δ_H_** = 9.8 | **δ_D_** = 15.3; **δ_P_** = 5.0; **δ_H_** = 9.1 |
| *CH* | 65.4 | 112 | **δ_D_** = 16.6; **δ_P_** = 0.1; **δ_H_** = 0.1 | **δ_D_** = 16.3; **δ_P_** = 0.1; **δ_H_** = 0.1 |

**MP wax: δ_D_** = 16.0; **δ_P_** = 1.6; **δ_H_** = 2.6

**Table S4.** Table showing solvent blend and ratio of each solvent required for CD solubilization.

| **Solvent blends** | **Ratio (v/v)** | **Solubility parameters 25°C** | **Ra (HSP distance) w.r.t MP wax** |
| --- | --- | --- | --- |
| Isododecane: IPA (*isoDD:IPA*) | 90:10 | **δ_D_** = 14.8; **δ_P_** = 0.7; **δ_H_** = 1.7 | 2.5 |
| Isododecane: hexanol (*isoDD:hex*) | 90:10 | **δ_D_** = 14.8; **δ_P_** = 0.6; **δ_H_** = 1.3 | 2.5 |
| Isohexadecane: IPA (*isoHD:IPA*) | 90:10 | **δ_D_** = 14.9; **δ_P_** = 0.7; **δ_H_** = 1.7 | 2.4 |
| *IPA: CH* | 50:50 | **δ_D_** = 16.3; **δ_P_** = 3.1; **δ_H_** = 8.3 | 6.5 |
| *tPA: CH* | 50:50 | **δ_D_** = 16.2; **δ_P_** = 2.5; **δ_H_** = 5.0 | 3.2 |
| *tPA* | - | **δ_D_** = 15.3; **δ_P_** = 5.0; **δ_H_** = 9.1 | 8.7 |

**S9:** **Evolution of Ra with progressive evaporation of solvent components**

**
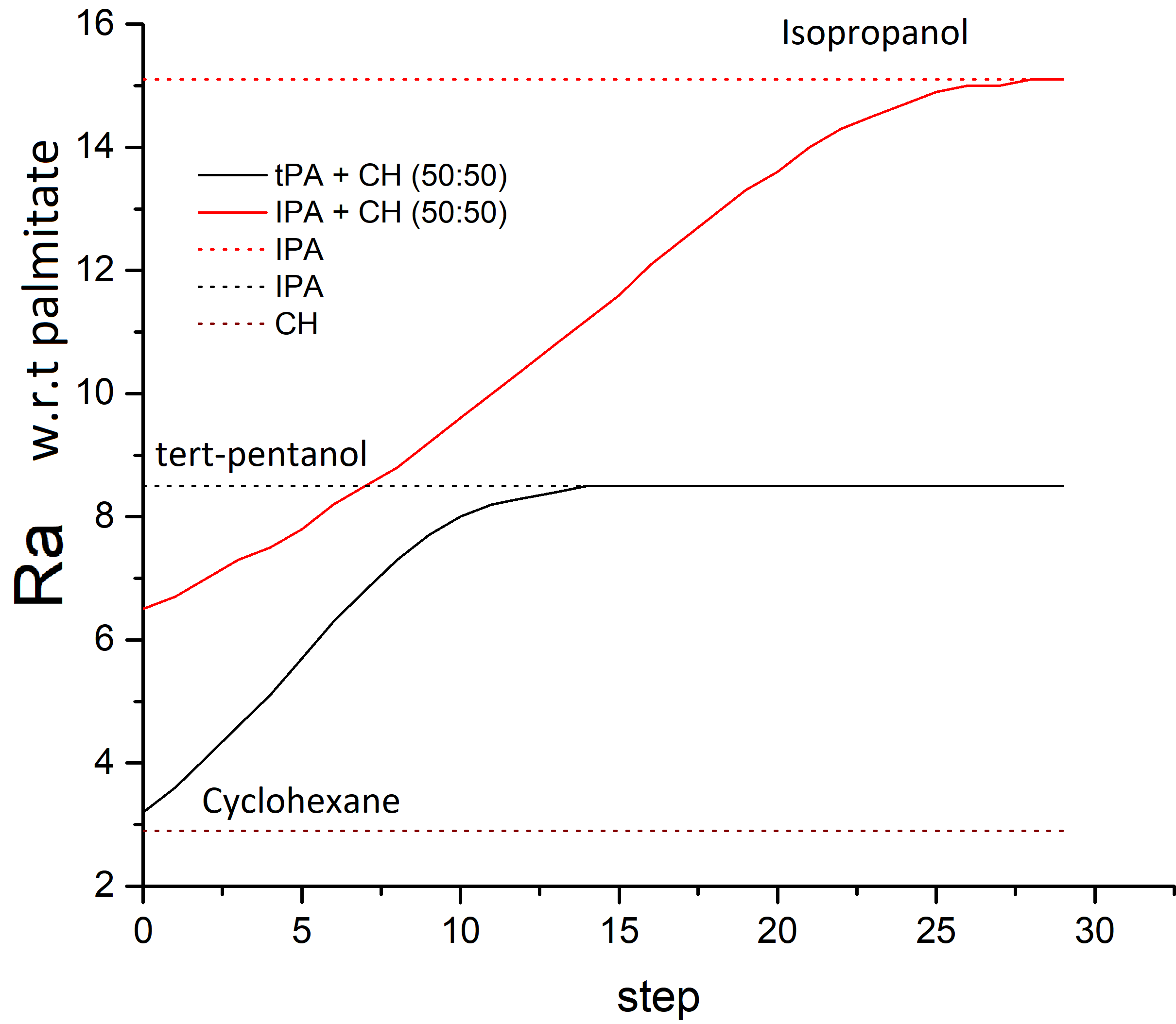
**

**Figure S6.** Evolution of **Ra** with progressive evaporation for different solvent blends as determined by simulation run in *HSPiP software*. (**black solid**) *tPA: CH (*50: 50); (**red solid**) *IPA: CH* (50:50); (**red dotted**) *IPA*; (**black dotted**) *tPA*; (**purple dotted**) *CH*.

**S10: Contribution of film thickness on optical performance**

Effectiveness of photo-protection can be determined by the radiation transmitting through film to the substrate. Film thickness greater than transport length for specific R.I. of material leads to decreased transmittivity and can be determined theoretically.[3] However, our previous work[2] show dependence of crystallinity (%) on re-crystallization kinetics (evaporation rate of solvent) which might influence optical properties of wax, *i.e.* known or reported value of refractive index. Due to unknown nature of optical properties of wax (refractive index), we experimentally show that film thickness ~5 µm is not sufficient to reduce transmission. We highlight that with the same film thickness, a structured film induces scattering which substantially reduces the fraction of radiation transmitting through the film to the substrate, thereby effectively enhancing photo-protection without the need for increase in film thickness.

Experimentally, for CD film, this was achieved by heating the film to high temperature of ~175°C at which point the wax melts on the quartz plates. Subsequently, the substrate is cooled down to room temperature as a result of which it partially crystallizes but from its melt, unlike re-crystallization from solvent evaporation. This procedure is schematically explained below in Scheme S3.


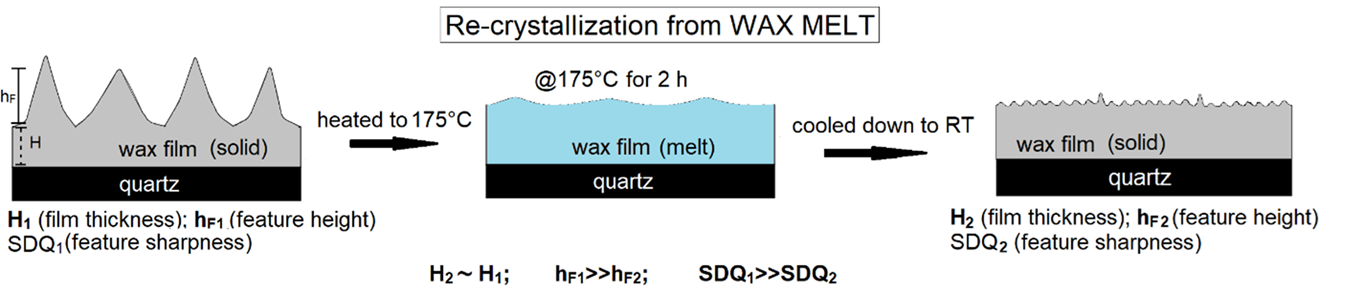


**Scheme S3.** Schematics showing the re-crystallization of wax from its molten state for obtaining flatter Candelilla films.

Re-crystallization of wax from its melt results in smoother surface which we believe is caused due to quenching of the wax film leading to partial re-crystallization and growth of smaller crystals. Figure S8 shows the differences in surface morphology and roughness of the film between film obtained from evaporative drying (Figure S7: a1) and the exact same film when re-crystallized from its melt. The average film thickness (**H**) remains in the range of 5 – 6µm before and after heating. However, the major difference is in the surface structuration where the roughness arising from vertically inclined crystal plates flattens out leading to smoother films with roughness at nanoscale. The feature height (**h_F_**) decreases from ~6.0 µm to ~0.25 µm and other details of roughness parameter is mentioned Table S5.


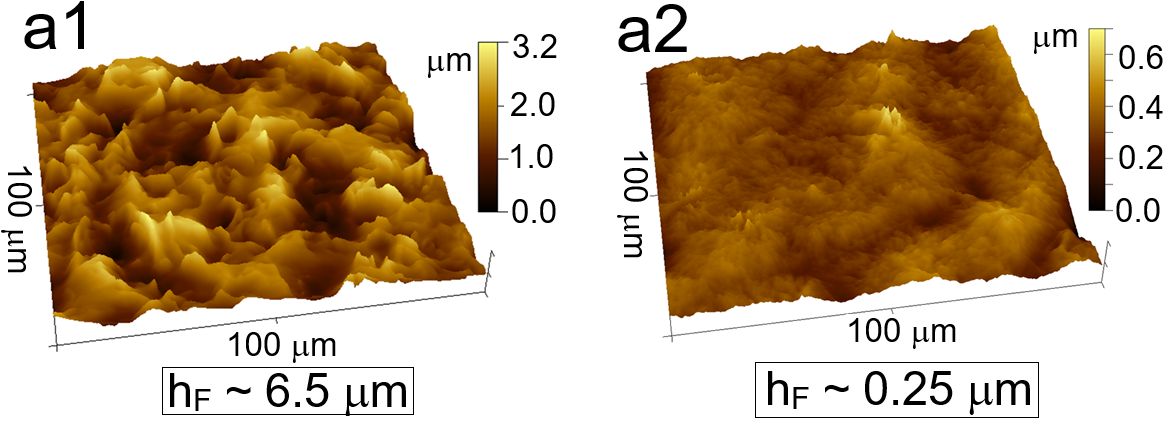


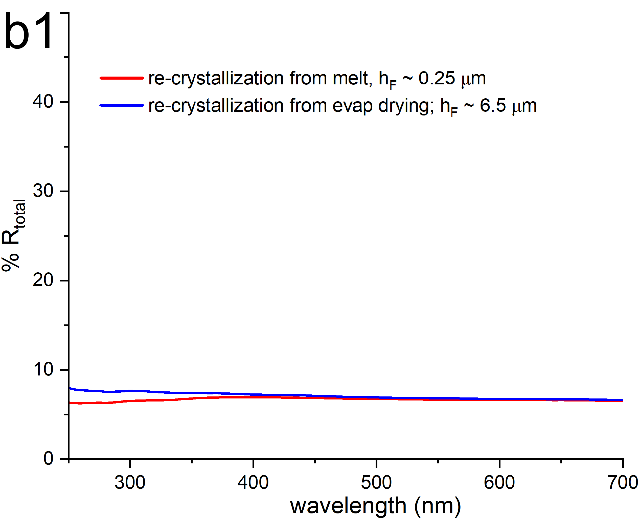

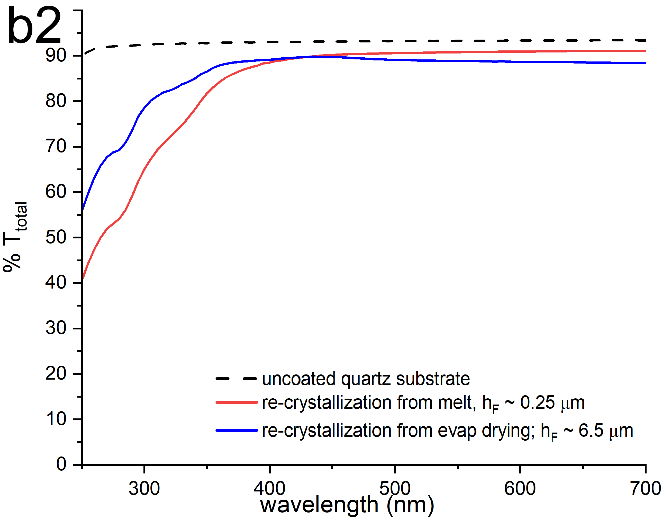


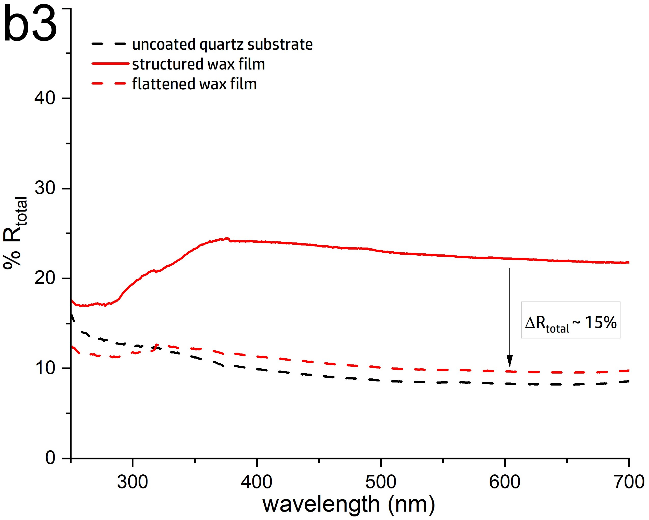


**Figure S7.** AFM images showing surface morphology of *Candelilla* wax films on quartz plates obtained on (**a1**) evaporative drying of drop-casted *Candelilla* wax solution in *isoDD:IPA* (90:10); (**a2**) after heating *Candelilla* wax films to 175°C and cooling at ambient conditions. (**b1 – b3**) Comparison of optical measurements of CD wax re-crystallized from evaporative drying and after re-crystallization from melt. (**b1 and b2**) **%R_total_** and **%T_total_** of structured film obtained from *isoDD:IPA* (before and after heating of films) respectively. (**b3**) **%R** for film obtained from *isoHD: IPA* (before and after heating of films).

**Table S5.** Roughness parameters for *Candelilla* wax film deposited from its solution in *isoDD:IPA* (at 37°C) and after re-crystallization from its molten state at 175°C

| **Structured wax film** |  | | **Roughness parameters** | | |
| --- | --- | --- | --- | --- | --- |
|  | **H (µm)** | **h_F_ (µm)** | | **SDQ** | **SAL (µm)** |
| Re-crystallization due to evaporative drying | 5.2 ± 0.85 | 6.8 ± 0.8 | | 1.8 ± 0.3 | 3.5 ± 0.40 |
| Re-crystallization from melt | 5.0 ± 0.15 | 0.25 ± 4.0 | | 0.09 ± 0.02 | - |

Corresponding optical reflectance and transmittance are shown in Figure S7: b1 and b2 where we observe no significant difference for shallow structured CD wax films particularly **%R_total_**. The spectral curve and **%T_total_** is close to that of the quartz substrate. However, film obtained from *isoHD:IPA* (90:10) with **%R_total_** ~ 30 - 40% in visible spectra show 15% drop in total reflectance (Figure S7: b3) indicating increase in fraction of radiation transmitting despite the same film thickness. This evidently shows that in visible spectra transmittivity is not influenced by thickness of *Candelilla* wax films and that it significantly reduces when the film of similar **H** is structured.

For better clarification in UV spectra, we considered MP films, re-crystallization film from melt was limited due to dewetting of wax at higher temperature, thereby, breaking the integrity of the film and exposing the substrate partially that typically would increase transmittivity.


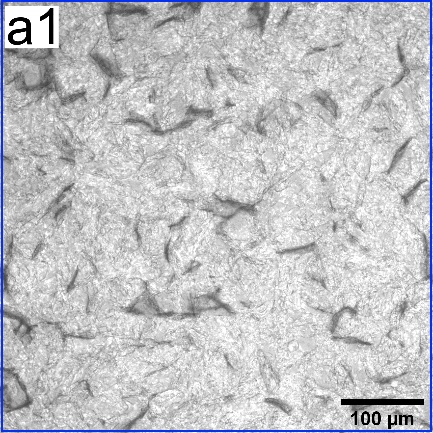

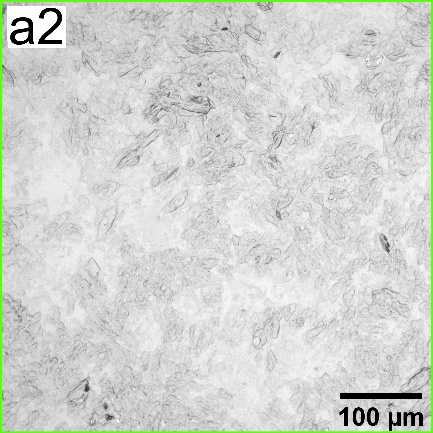

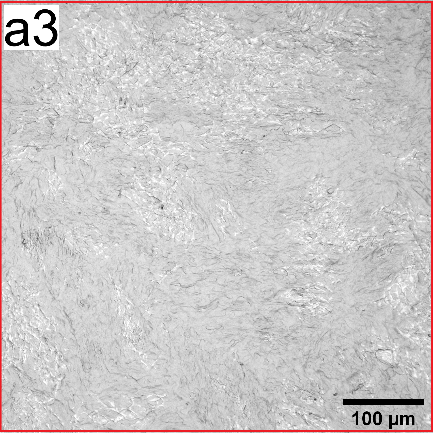


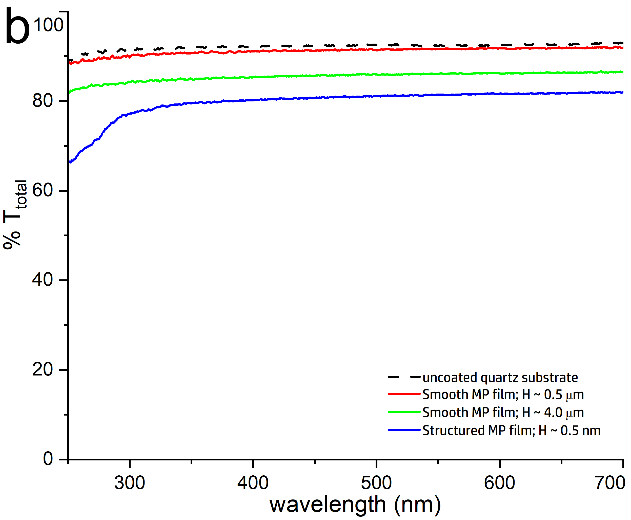


**Figure S8.** Optical transmittivity as measured by UV-Vis Spectrophotmeter for MP films coated at different conditions.

To overcome this, we obtained several films with different thicknesses and surface roughness by drop casting and spin coating. Drop casting from *isoDD:IPA* (90:10 v/v) leads to flatter films without any 3D structuration due to better solubility of MP wax in the solvent mixture of *isoDD:IPA* (Hansen Solubility Parameter Distance, **Ra** – 2.6 vs 6.2 mentioned Table S3 and S4 ) and slower evaporation kinetics of the solvents. From graphical representation in Figure S8, we observe **%T_total_** of spin coated film at **H**~ 0.9 µm is similar to that of quartz substrate while there is a decrease of ~5% for film with **H**~ 4 µm. In contrast, comparison of 3D structured (blue solid line) and flatter (red solid line) spin coated films with **H**~ 0.8 µm shows a decrease of ~10% for 350 – 700 nm. Interestingly, we observe a monotonic decrease from 250 – 350 nm for 3D structured film.

From these controlled experiments, we believe that photo-protectivity arising out of film thickness of wax (investigated in this work) is minimal and that presence of structures substantially improves it without the need to increase the thickness (*i.e.* deposit more amount of wax).

**S11: Atomic Force Microscopy of MP films on SC**


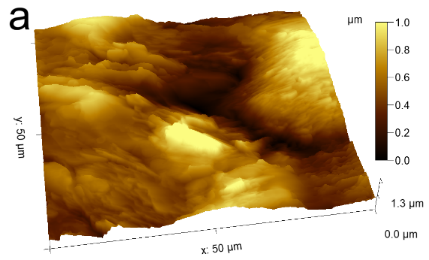

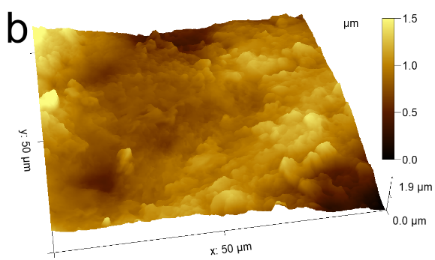

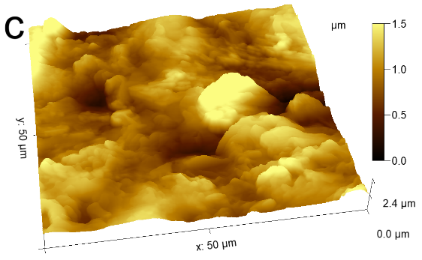


**Figure S9.** Surface imaging of MP wax films coated on SC with AFM revealing roughness at sub-micron length scale. Films obtained on deposition of MP wax from its solution in (**a**) *IPA:CH*; (**b**) *isoDD*; (**c**) *CH*.

**S12:** **Wettability (water contact angle) of casted biomimetic films**

**Table S6.** Table showing water contact angle on biomimetic films obtained at different casting conditions.

| **Substrate** | **Wax films** | **Coating conditions** | **Morphology** | **Water contact angle (°)** |
| --- | --- | --- | --- | --- |
| Quartz plates | Candelilla | *isoDD:IPA (37°C)* | Single – scale | 100 ± 2 |
|  |  | *isoHD:IPA (37°C)* | Single – scale | 105 ± 5 |
|  | Myristyle Palmitate | *tPA* | Dual – scale | 100 ± 3 |
|  |  | *tPA:CH* | Single – scale | 105 ± 1 |
| PMMA plates  (smooth) |  | *isoDD* | Dual – scale | 105 ± 1 |
|  |  | *CH* | Dual – scale | 103 ± 2 |
| PMMA plates  (textured) |  | *isoDD* | - | - |
|  |  | *CH* | - | - |
| *Stratum Corneum* |  | *isoDD* | Dual – scale | 104 ± 3 |
|  |  | *CH* | Dual – scale | 102 ± 2 |

**References:**

[1] M.E. Ginn, C.M. Noyes, E. Jungermann, The contact angle of water on viable human skin, J Colloid Interface Sci 26 (1968) 146–151. https://doi.org/10.1016/0021-9797(68)90306-8.

[2] A. Das, L. Polacchi, C. Courreges, J.-Y. Fouron, L. Tournier-Couturier, L. Billon, G.S. Luengo, Evaporative Drying Induced Self-Assembly of Epicuticular Wax: A Biomimetic Approach in Tuning Surface Roughness, Langmuir 40 (2024) 7581–7594. https://doi.org/10.1021/acs.langmuir.4c00205.

[3] V. Hwang, A.B. Stephenson, S. Barkley, S. Brandt, M. Xiao, J. Aizenberg, V.N. Manoharan, Designing angle-independent structural colors using Monte Carlo simulations of multiple scattering, Proc Natl Acad Sci U S A 118 (2021). https://doi.org/10.1073/pnas.2015551118.
